# Phylogenomic insights into LA-MRSA from Argentine pig farm environments: novel *OptrA* variant and regional emergence of an ST9 lineage co-circulating with international CC398 lineages

**DOI:** 10.3389/fmicb.2025.1662779

**Published:** 2025-10-09

**Authors:** María José González, Julián Parada, Enrique Blasko, Ezequiel Jorge Sosa, Geehan Suleyman, Jagjeet Kaur, Gina Maki, Tyler Prentiss, Maite Corti Isgro, Natalia Pereyra, Rocío González, Agostina Dagatti, Paula Gagetti, Fernanda Pacharoni, Ricardo Toselli, Davor Nicolas Martinovic Bolia, Cesar Bonetto, Carolina Del Bo, Carina Porporatto, Darío Fernández Do Porto, Laura Decca, José Luis Bocco, María Valeria Amé, Alejandra Corso, Alicia Isabel Carranza, Marcus Zervos, Claudia Sola

**Affiliations:** ^1^Departamento de Bioquímica Clínica, Facultad de Ciencias Químicas, Universidad Nacional de Córdoba and Centro de Investigaciones en Bioquímica Clínica e Inmunología (CIBICI) CONICET, Córdoba, Argentina; ^2^Departamento de Patología Animal, Facultad de Agronomía y Veterinaria, Universidad Nacional de Río Cuarto, Río Cuarto and Instituto de Ciencias Veterinarias (INCIVET), CONICET, Río Cuarto, Argentina; ^3^Laboratorio de Sistemas, Universidad Tecnológica Nacional, Regional Buenos Aires, Ciudad Autónoma de Buenos Aires, Argentina; ^4^Division of Infectious Diseases, Henry Ford Hospital, Detroit, MI, United States; ^5^The Global Health Initiative, Henry Ford Health, Detroit, MI, United States; ^6^Laboratorio de Microbiología, Clínica Regional del SUD-Río IV, Río Cuarto, Argentina; ^7^Servicio Antimicrobianos, Instituto Nacional de Enfermedades Infecciosas (INEI)-ANLIS “Dr. Carlos G. Malbrán”, Ciudad Autónoma de Buenos Aires, Argentina; ^8^Unidad de Microbiología, CEPROCOR, Ministerio de Producción, Ciencia e Innovación Tecnológica de Córdoba, Córdoba, Argentina; ^9^Laboratorio de Microbiología, Hospital Guillermo Rawson, Córdoba, Argentina; ^10^Instituto Multidisciplinario de Investigación y Transferencia Agroalimentaria y Biotecnológica (IMITAB) CONICET and Universidad Nacional de Villa María, Instituto Académico Pedagógico de Ciencias Básicas y Aplicadas, Villa María, Argentina; ^11^Facultad de Ciencias Exactas y Naturales, Departamento de Química Biológica, Universidad de Buenos Aires, Buenos Aires, Argentina

**Keywords:** livestock-associated methicillin-resistant *Staphylococcus aureus*, pig farm environment, clonal complexes CC398 and CC1/ST9, antimicrobial resistance, Argentina, *OptrA* variant

## Abstract

**Introduction:**

*Staphylococcus aureus* (SA) colonizes both humans and animals. The spread of livestock-associated methicillin-resistant *S. aureus* (LA-MRSA) in farms and the environment poses a public health concern. While CC398 is globally predominant, regional variation exists, and data from Argentina remain scarce. This study investigated the presence, antimicrobial resistance (AMR), and genomic features of pig-associated MRSA in central Argentina, including transmission dynamics and the global phylogenetic context of LA-MRSA lineages CC398 and CC1/ST9.

**Methods:**

Between February and March 2022, 41 MRSA isolates were recovered from 50 fecal and effluent samples collected from 8 of 10 (80%) farrow-to-finish pig farms in Córdoba province, Argentina. Phenotypic susceptibility was assessed by disk diffusion and Vitek2 (CLSI 2023). Genotypic characterization included PCR for antimicrobial resistance genes (ARGs) and the immune evasion cluster (IEC), as well as PFGE, *spa* typing, SCC*mec* typing, and MLST. Twenty-four isolates underwent whole-genome sequencing (Illumina NovaSeq 6000) for resistome, virulome, and phylogenomic analysis.

**Results:**

Antimicrobial use was extensive, with most farms reporting use of more than seven drug classes and up to 18 compounds in the previous six months. Two predominant IEC¯ LA-MRSA lineages were identified, mostly associated to SCC*mecV*: CC398 (53.7%, n:22), comprising ST398 (n:17, *spa*-types *t034/t571*) and ST8814 (n:5, *t571*); and CC1/ST9 (41.5%, n:17, *spa*-t16964). One IEC+ CA-MRSA lineage (CC8/ST72, n:2, SCC*mecI*Va) was also detected. All isolates were multidrug-resistant (MDR), with LA-MRSA showing broader resistance across 4–7 antimicrobial classes. Multiple ARGs matched phenotypic resistance, except *optrA*, found in ST398 (n:5) and ST9 (n:1) despite borderline linezolid MICs (2–4 µg/mL), representing the first report of *optrA* in *S. aureus* in Argentina. WGS revealed a novel optrA variant (*OptrA*/FDKFP), likely linked to a mobile genetic element. Phylogenetic analysis revealed the local emergence of a distinct ST9 clade and multiple introductions of CC398 within internationally recognized subgroups (C6/AAP and C6/EP4).

**Discussion:**

These findings underscore pig farms as emerging reservoirs of multidrug-resistant MRSA both locally and globally, reinforcing the need for targeted surveillance and control strategies at the animal–human interface.

## 1 Introduction

*Staphylococcus aureus* (SA) is a common commensal in humans and animals, including livestock, and a major opportunistic pathogen responsible for infections ranging from skin lesions to fatal bacteremia (Park and Ronholm, 2021). Beyond its virulence, its significance lies in its remarkable ability to acquire antimicrobial resistance, particularly methicillin resistance (MRSA), and to spread across healthcare (HA), community (CA), and livestock-associated (LA) settings (Park and Ronholm, 2021; Kasela et al., 2023). In 2019, SA was the leading bacterial cause of death globally and the second in antimicrobial resistance (AMR)-related mortality, ranking first in the Americas. It also showed the sharpest increase in AMR-attributed deaths from 1990 to 2021 and is classified by WHO as a high-priority pathogen (GBD 2019 Antimicrobial Resistance Collaborators, 2022; GBD 2021 Antimicrobial Resistance Collaborators, 2024; Antimicrobial Resistance Collaborators, 2022, 2023; Sati et al., 2025).

Within the One Health framework, AMR is driven by antimicrobial use in human medicine, agriculture, and animal production, particularly pig farming, and by environmental stressors such as heavy metals, which facilitate the persistence and spread of AMR-bacteria and AMR-genes (ARGs) across human-animal-environmental sectors (Wang et al., 2023; Khairullah et al., 2023). In 2015, Argentina adopted the One Health approach and launched a national AMR strategy. In August 2022, legislation was passed to regulate antibiotic use and sales in both human and veterinary contexts.^1^ In pig production, antimicrobials are widely used for treatment and prevention. Antibiotic growth promoters have been banned in several countries, including Argentina since 2024 (SNSyCAA/Res.445, 2024), but remain permitted elsewhere (Muurinen et al., 2021).

Although SA is endemic worldwide, the emergence of high-risk clones (HRCs), defined by enhanced transmissibility, virulence, and/or multidrug resistance (MDR), has accelerated its global spread (Aanensen et al., 2016). MDR HA-MRSA clones have circulated in hospitals since 1959. From the 1980s, distinct CA-MRSA clones emerged with regional variation and now also cause hospital-onset infections (Egea et al., 2014; Lakhundi and Zhang, 2018; Barcudi et al., 2020). LA-MRSA is a significant zoonotic pathogen of growing global public health concern. It was first isolated from bovine mastitis in 1972, and zoonotic transmission from pigs (ST398 lineage) was first reported in the Netherlands in 2005 (Wang et al., 2023). Today, pigs are recognized as important reservoirs of MDR LA-MRSA, primarily acting as asymptomatic carriers colonized on skin and mucosa. Direct animal contact is the main transmission route on farms, while contaminated environments and animal products further amplify dissemination (Khairullah et al., 2023; Wang et al., 2023). MRSA positivity in pig farms varies by region; in countries such as Denmark and the Netherlands, over 75% of farms are MRSA-positive (Sieber et al., 2018; EFSA and ECDC, 2023).

While the prevalence of MDR LA-MRSA remains lower than that of HA- or CA-MRSA, infections are increasing, sometimes in individuals without livestock contact, raising public health concerns, particularly in regions with intensive pig production (Chen et al., 2020; Matuszewska et al., 2022; Chen et al., 2023; Khairullah et al., 2023; Campana-Burguet et al., 2025) Human-to-human transmission has been reported, especially from farm workers to household members, and occasionally in healthcare settings (Avbersek et al., 2021; Crespo-Piazuelo and Lawlor, 2021; Wang et al., 2023). Reverse zoonosis from humans to pigs has also been documented (Narvaez-Bravo et al., 2016; Sahibzada et al., 2017).

Clonal complex (CC) 398 is the dominant LA-MRSA lineage worldwide, particularly in Europe, but has also been reported in the Americas, Asia, and Oceania (Sahibzada et al., 2017; Kruger-Haker et al., 2023; Khairullah et al., 2023; Gagetti et al., 2023b). Another key lineage, ST9/CC1, predominates in Asia and is emerging in the USA and Europe (Kasela et al., 2023; Wang et al., 2023; Khairullah et al., 2023). Both lineages commonly exhibit MDR and have been linked to zoonotic transmission, including cases without direct livestock contact (Chen et al., 2020; Jin et al., 2020; Chen et al., 2023; Kruger-Haker et al., 2023). Of particular concern is their resistance to oxazolidinones, last-resort drugs for Gram-positive infections in humans. Three transferable resistance genes, *cfr*, *optrA*, and *poxtA*, have been primarily identified in livestock-associated staphylococci and are notable for their cross-resistance, mobility, and co-selection potential (Schwarz et al., 2018; Schwarz et al., 2021; Timmermans et al., 2021).

Phylogenetic evidence suggests that LA-CC398 originated from a human-associated methicillin-susceptible *Staphylococcus aureus* (MSSA) ancestor that acquired *tet* (M) and SCC*mec* elements after transmission to livestock, while losing the immune evasion cluster (IEC). IEC reacquisition facilitates re-adaptation to human, illustrating the bidirectional nature of zoonotic transmission. Notably, IEC gain/loss drives host shifts not only in CC398 but also in CC1/ST9, CC1, and CC5 (Matuszewska et al., 2022; Wang et al., 2023; Chen et al., 2020; Yu et al., 2021). Accordingly, CC398 comprises two major clades: a livestock-associated (LA) clade [IEC^−^, *tet(M)*^+^, mostly MRSA] and a human-associated (HuA) clade [IEC^+^, *tet(M)*^−^, mostly MSSA] (Wang et al., 2023). A recent study of over 3,000 CC398 genomes revealed geographically structured phylogroups across Asia, Europe, and the Americas, each with distinct resistance and virulence profiles (Fernandez et al., 2024), emphasizing the need for regional genomic surveillance of LA-SA.

In Argentina, SA infections have increased by 23.4% over the past decade, primarily driven by community-onset MSSA with rising macrolide and gentamicin resistance, particularly the CC398-t1451-*erm(T)*^+^ clone (Di Gregorio et al., 2023; Barcudi et al., 2024). Although MRSA incidence remained stable and mostly HA-related, a shift from ST5-I to diverse clones, CC5 (ST5-IV, ST100-IV), CC30 (ST30-IV), and minor lineages such as CC97 (ST97-IV) and CC8 (ST8-IV, ST72-IV); has been observed (Barcudi et al., 2024).

Pig farming is a key sector in Latin America, with pork consumption increasing by 57% between 2005 and 2022 (Roppa et al., 2024). Brazil and Mexico lead the sector, followed by Argentina and Colombia, which together contribute 14% of global exports. In Argentina, production is concentrated in Córdoba and Buenos Aires (24% each of national stock), and is highly fragmented: just 2% of farms (>2,500 sows) produce half the slaughter pigs (SAGyP, 2023). Despite the known role of pigs as MRSA reservoirs, data on circulating lineages in Argentine farms remain limited (Gagetti et al., 2023b).

This study aimed to: (i) identify and characterize MRSA strains from pig farm environments (feces/sludge and effluents) in the central region of Argentina (Córdoba Province); and (ii) characterize LA-MRSA lineages CC1/ST9 and CC398 using whole-genome sequencing, with a focus on resistome and virulome. Potential MRSA transmission within and between farms was also assessed. Additionally, a comparative phylogenetic analysis was performed using publicly available genomes from the Americas, Europe, and Asia to contextualize Argentine isolates globally.

## 2 Materials and methods

### 2.1 Selection of pig farms and sampling

A cross-sectional study was conducted between February and March 2022 on 10 intensive indoor farrow-to-finish pig farms located within a 100 km radius of Río Cuarto city, Córdoba, Argentina (Table 1). Farms were selected based on proximity to the National University of Río Cuarto (UNRC) and existing connections with farmers and veterinarians, constituting a convenience sample. Farmers were informed of study aims and sampling protocols at the time of visit; none refused participation. Ethical approval was granted by the UNRC Central Bioethics Committee (Resolution 376/22, CS-UNRC). Each farm was visited by two veterinarians, who asked farm owners (Yes/No) whether antibiotics had been used for any purpose (treatment, prevention, or growth promotion) in the previous 6 months, and to specify the antibiotics used according to their records. Five environmental samples were collected per farm from high-traffic, feces-contaminated areas, covering different production stages: breeding stock, lactating piglets, nursery (40 ± 4 days of age), finishing pigs (120 ± 4 days of age), and effluents. Environmental samples were collected using absorbent overshoes walked in an “X” pattern per area, with two overshoes per location placed in a sterile polyethylene bag. Effluent samples (50 mL) were taken from the pipe leading to the treatment lagoon. Samples were stored at 4°C and processed within 8 h.

**Table 1:** MRSA status of fecal and effluent samples and molecular characteristics from 10 pig farms in Córdoba Province, central Argentina.

Farm ID (n)^a^	Production stage (category/pen) of environmental fecal samples					
	Breeding stock	Lactating piglets	Nursery (40 ± 4 days)	Finishing pigs (120 ± 4 days)	Swine production effluents	Total
	MRSA status^b^					
1 (2500)	+*	−	(+)*	+*, +*	(+), +*	+
2 (180)	−	−	(+**), +**	+**, +**	(+)**, +**	+
3 (200)	−	−	(+), +	(+), +	−	+
4 (1000)	(+, +)	−	−	−	−	+
5 (500)	(+)	(+), +	(+), +	(+), +	(+), +	+
6 (750)	−	−	(+)**^c^, +**^c^	+**^d^	(+)**^d^	+
7 (200)	(+, +)	(+)	−	(+)	(+)*	++
8 (400)	−	−	−	−	−	−
9 (150)	−	(+, +)	(+)	(+), +	−	+
10 (2000)	−	−	−	−	−	−
% positive	50 (4/8)	37.5 (3/8)	75 (6/8)	87.5 (7/8)	62.5 (5/8)	8

^a^Farm ID: Identification number of each farm and (n), the number of sows per farm (1–10). ^b^MRSA status: “+” indicates MRSA-positive, with “+” representing one MRSA strain recovered and “+,+” indicating two MRSA strains recovered. “(+)” denotes one sequenced MRSA strain, while “(+, +)” indicates two sequenced MRSA strains. Background colors represent MRSA lineages: dark gray, CC398 MRSA; light gray, ST9/CC1 MRSA; white, ST72/CC8 MRSA. *optrA-positive; **fosB-positive. MRSA strains carrying ^c^SCC*mec* III-like, ^d^SCC*mec* V-like.

### 2.2 Sample processing and microbiology

Overshoe samples were diluted 1:10 (10 mL/g) in PBS, homogenized, and centrifuged (600 rpm, 2 min); supernatants were further diluted 1:10 in PBS. Effluent samples followed the same procedure. One milliliter of each dilution was enriched in 9 mL tryptic soy broth with 6.5% NaCl and incubated overnight. Then, 100 μL were plated on CHROMagar™ Staph aureus (CHROMagar Microbiology, France). Presumptive SA colonies (1–2 per plate) were confirmed by morphology, standard biochemical tests, and PCR for the *nuc* gene.

### 2.3 Susceptibility testing

Antimicrobial susceptibility was assessed by disk diffusion and Vitek2 AST-P653 cards (bioMérieux, France) according to CLSI guidelines (Clinical and Laboratory Standards Institute, 2023). Antibiotics tested by disk diffusion (disk content in brackets) included: cefoxitin (FOX, 30 μg), penicillin (PEN, 10 U), gentamicin (GEN, 10 μg), erythromycin (ERY, 15 μg), clindamycin (CLI, 2 μg), tetracycline (TET, 30 μg), minocycline (MIN, 30 μg), chloramphenicol (CMP, 30 μg), trimethoprim/sulfamethoxazole (SXT, 1.25/23.75 μg), rifampicin (RIF, 5 μg), ciprofloxacin (CIP, 5 μg), and linezolid (LZD, 30 μg). Vancomycin (VAN) was evaluated by minimal inhibitory concentration (MIC) with Vitek2. For *optrA*-positive MRSA, LZD susceptibility was evaluated by both Vitek2 and E-test (bioMérieux, France). *S. aureus* ATCC 29213 and ATCC 25923 were used as quality control strains for MIC and disk diffusion testing, respectively. Antimicrobial susceptibility results were interpreted according to CLSI guidelines (Clinical and Laboratory Standards Institute, 2023). Fosfomycin (FOS) was not tested, following EUCAST recommendations (EUCAST, 2024), as no clinical breakpoints are available and current methods lack reliable correlation with clinical outcomes. Multidrug resistance (MDR) was defined as resistance to ≥3 antimicrobial (ATM) classes (Magiorakos et al., 2012). PCR was used to detect *mecA*, *mecC*, and additional resistance genes (Benito et al., 2014) based on phenotypic profiles (Table 2). *optr*A, *fos*B, and *tet*(T) genes, identified in sequenced genomes, were also screened by PCR in all MRSA (Supplementary Table S1 for primers).

**Table 2:** Characteristics of 41 MRSA strains isolated from fecal and effluent samples in pig farm environments in central Argentina.

CC/n, %^a^	ST/, n, % ^a^	FARM	PFGE type/n, % ^a^	PFGE Subtype/no.(%)^b^	CC398 *sau1-hsdS1*^c^	RIDOM *spa* type/no. (%)^b^	SCC*mec* no. (%)^b^	*agr* type	IEC genes^c^			Resistance phenotype^d^ (no. of isolates)	Resistance genotype^e^/(no. of isolates)
									*scn*	*sak*	*chp*		
CC398 22, 53.7	398/, 17, 41.5	1, 2, 6, **7**	NT/17 41.5	NT/17 (100)	+	*t*034: 11(64,7), *t*571: 6 (35.3)	V: 13 (76.4), V-l^f^: 2(11.8), III-lg: 2 (11.8)	1	─	─	─	**1-**6 AC: FOX, PEN, TMS, ERY, CLI-c, TET, MIN, CMP, CIP (10)	*dfrG*, *erm*(C), *lnu*(B), isa(E), *fexA*, tet(M), *fosB*/(4)
													*dfr*G, *erm*(C), *fexA*, *tet*(M), *tet*(K), *optrA*(5)/(6)
												**2-**7 AC: FOX, PEN, TMS, ERY, CLI-c, TET, MIN, CMP, GEN, CIP (6)	*dfrG, erm*(C), *erm*(T), *lnu*(B), *isa*(E), *fexA, aacA-aphD, tet*(M), *tet*(K)(*2*), *tet*(L), *fosB*/(6)
												**3-**4C: FOX, PEN, TMS, CLI-l, TET, MIN (1)	*dfrG, lnu*(B), *isa*(E), *tet*(M), *tet*(L)/(1)
	ST8814^h^/5, 12.2	9	NT/5 12.2	NT/9 (100)	+	*t*571: 5 (100)	V: 5 (100)	1	─	─	─	**2-**7 AC: FOX, PEN, TMS, ERY, CLI-c, TET, MIN, CMP, GEN, CIP (5)	*dfr*G, *erm*(C), *erm*(T), *lnu*(B), isa(E), *fexA*, *aacA-aphD*, *tet*(M), *tet*(K)(4), *tet*(L)/(5)
CC1 17, 41.5	9/ 17, 41.5	5,3,7	X/17 41.5	X3/16 (94.1), X4/1 (5.9)	─	*t*16964: 16 (94.1) *t*NA^i^:1 (5.9)	V: 17 (100)	2	─	─	─	**4-**4 AC: FOX, PEN, ERY, CLI-c, CMP, CIP (13)	*erm*(C), *vga*(A)(*2*), *fexA*/(12)
													*erm*(C), *fexA, optrA/(1)*
												**5-**4 AC: FOX, PEN, CLI-l, CMP, CIP (2)	*vga*(A), *fexA/*(2)
												**6-**5 AC: FOX, PEN, ERY, CLI-c, TET, MIN, CMP, CIP (1)	*erm*(C), *vga*(A), *fexA, tet*(T), *tet*(L)/(*1*)
												**7-**4 AC: FOX, PEN, CLI-l, TET, MIN, CMP, CIP (1)	*vga*(A), *fexA, tet*(T), *tet*(L)/(*1*)
CC8 2, 4.8	72/2 4.8	4	R/2, 4.8	R26/2 (100)	─	*t*3092: 2 (100)	IVa: 2 (100)	3	+	+	+	**8-**3 AC: FOX, PEN ERY, TET (2)	*msr*(A),*mph*(C), *tet*(K)/(2)

CC, Clonal Complex; ST, Sequence Type, PFGE type/subtype, Pulsed Field Gel Electrophoresis type and subtypes; RIDOM *spa* type: staphylococcal protein A (*spa*) type assigned through the RIDOM databases (http://spaserver.ridom.de); SCC*mec*: Type of Staphylococcal Cassette Chromosome *mec*; *agr* type, type of accessory gene regulator allotype. ^a^n, %: number and % of total MRSA (n: 41) of each CC or ST or PFGE type (NT: not typeable by SmaI-PFGE). ^b^no. (%), number and % of strains with this molecular characteristic (PFGE subtype or *spa*-type or SCC*mec* type) belonging to each ST. ^c^CC398 *sau1-hsdS1* gene (Stegger et al., 2011) and IEC genes (immune evasion complex genes: *scn, sak* and *chp*): + positive or *─* negative by PCR. ^d^Resistance phenotype is indicated as follows: Pattern (number: 1–8)—number of resistant antimicrobial classes (AC) and specific antimicrobial resistances detected in each pattern. The tested antimicrobials include cefoxitin (FOX), penicillin (PEN), trimethoprim/sulfamethoxazole (SXT), erythromycin (ERY), clindamycin (CLI-c: constitutive macrolide-lincosamide-streptogramin B resistance; CLI-l: lincosamide-only resistance), tetracycline (TET), minocycline (MIN), chloramphenicol (CMP), gentamicin (GEN), and ciprofloxacin (CIP). ^e^Resistance genotype: The presence of 20 antibiotic resistance genes: *tet*(M)/(L)/(K)/(T)*, erm*(A)/(B)/(C)/(T)*, msr*(A)*, mph*(C)*, lnu*(A)/(B)*, vga*(A)*, isa*(E)*, optrA*, *fexA*, *aacA-aphD* or *aac(6′)-aph(2″)*, *dfrG*, *dfrA*, and *fosB,* was analyzed by PCR. Detected genes are indicated; the number of positive isolates is shown in parentheses when not all isolates carried the gene. ^f^SCC*mec* V-like: positive for *ccrC1* gene (*ccr* gen complex 5) and class C2 *mec* gene complex and harbored additional *ccrA1/ccrB1* genes. *^g^*SCC*mec* III-like: positive by for class A *mec* gene complex *and ccr3 gene complex*, but negative for SCCHg (*mer* operon and J region in SCC*mercury*; Chongtrakool et al., 2006) and J1 region of SCC*mec* III (Milheirico et al., 2007). ^h^New ST detected in this study. ^i^*spa* type (07–23–02-12-23-12-23-34) not assigned (NA) but related to *t*16964 (07–23–02-12-23-34).

### 2.4 Molecular typing

All MRSA isolates underwent SCC*mec* typing, PFGE (SmaI), and *spa* typing. *spa* types were used to infer MLST based on published correlations (Barcudi et al., 2020; Barcudi et al., 2024). Isolates were PCR-screened for *agr* types and IEC genes (*scn, chp, sak, sea, sep*), and for the CC398 marker *sau1-hsdS1* (Stegger et al., 2011).

### 2.5 Whole-genome sequencing and bioinformatics

A representative subset of 24 isolates (11 CC398, 11 ST9, 2 ST72) was selected for WGS. Isolates were chosen from each of the eight MRSA-positive farms, prioritizing those from different animal categories, effluents, and SCC*mec* variants identified by PCR. Additionally, the two unique CA-MRSA ST72 isolates were sequenced to assess their genomic characteristics, and two LA-MRSA isolates of the same ST and from the same animal category were included to evaluate Single Nucleotide Polymorphisms (SNPs) differences among closely related isolates (IID7/ST9 and ID9/ST8814). Additionally, nine MRSA isolates from Córdoba (2019–2022), five CC398 and four CC1/ST9, from various sources were included for comparison. Public genomes (n: 165, CC398: 98, and CC9: 67) from NCBI and prior studies (Zhou et al., 2018; Ji et al., 2021; Jiang et al., 2021; Randad et al., 2021; Yu et al., 2021; Li et al., 2022) were also analyzed. Sample name, accession number or Bioproject, country of origin, host, phylogenetic data, genotype and resistome are listed in Supplementary Tables S2.1, S2.2.

WGS was performed at the Genome Sciences Core Lab, Wayne State University, (Detroit, MI, United States), using Illumina NovaSeq 6,000 (150 bp paired-end reads). Reads were trimmed with Trimmomatic v0.39 (Bolger et al., 2014) and assembled using SPAdes v3.14 (Bankevich et al., 2012). Assembly quality was checked with QUAST v5.2.0 (Mikheenko et al., 2023). All assemblies had <200 contigs and N50 > 40,000 bp (Supplementary Table S2.3). Annotation was done with Prokka (Seemann, 2014). Species ID was confirmed using KmerFinder v3.2 (Larsen et al., 2014); STs were assigned via PubMLST. SCC*mec* and *spa* types were determined using SCC*mec*Finder v1.2 and spaTyper v1.0, respectively, Center for Genomic Epidemiology (CGE) (Bartels et al., 2014). Detailed genomic analyses of antimicrobial resistance genes (ARGs), mobile genetic elements (MGEs), virulence genes and phylogenetic analysis are provided in Supplementary material 1 (Darling et al., 2004; Li and Durbin, 2009; Li et al., 2009; Rissman et al., 2009; Jensen et al., 2010; McKenna et al., 2010; Li et al., 2011; Cingolani et al., 2012; Lozano et al., 2012; Stamatakis, 2014, 2015; Cook and Andersen, 2017; van der Graaf-van Bloois et al., 2021) and summarized in each corresponding figure.

### 2.6 Phylogenetic analysis

Three maximum-likelihood (ML) trees were constructed from core genome SNPs, which were identified using the approach of Aanensen et al. (2016) based on 26 fully annotated *S. aureus* reference genomes to define the core genome (see Supplementary material 1 for details). The first ML tree included all 24 MRSA isolates from pig environments, using SA LGA251 (FR821779) as reference. Two lineage-specific ML trees (CC398 and ST9/CC1 with S0385 and QD-CD9 as references, respectively) included our MRSA isolates, public genomes (CC398, n: 98, LA: 68 and HuA: 30, Matuszewska et al., 2022; ST9/CC1, n: 67), and additional local genomes (CC398 n: 5 and ST9/CC1 n:4) from Córdoba (2019–2022). The latter were selected from SA strains recovered in ongoing, unpublished One Health surveillance studies (humans, rivers, animals, and food) in central Argentina (Supplementary Table S2.2).

To explore farm-level transmission, two additional SNP trees (CC398 and ST9/CC1, n: 11 each) were generated with CSI Phylogeny v1.4 (Kaas et al., 2014) with lineage-specific reference genomes (S0385/ST398 and QD-CD92/ST9). Given the lack of consensus on SNP cut-offs for transmission inference (<15 to <50 SNPs), a 40-SNP threshold was used to define closely related strains or transmission cluster (Price et al., 2014; Long et al., 2014; Schurch et al., 2018; Bouchami et al., 2020; Randad et al., 2021).

### 2.7 Statistical analysis

Analyses were performed using SPSS v26.0 and InfoStat.^2^ Differences between proportions were assessed using chi-squared tests, with *p* < 0.05 considered significant. Comparative analysis of ARGs counts within each lineage (ST9/CC1, ST72/CC8, ST398-ST8814/CC398) was performed using Shapiro–Wilk, one-way ANOVA, and Tukey’s test in R; plots were generated with ggplot2.

## 3 Results

### 3.1 Farm characteristics and antimicrobial usage

Among 10 pig farms surveyed, four were small (<300 sows), two medium (301–700 sows), and four large (>700 sows) (Table 1). Farm-reported ATM use over 6 months revealed predominant drug classes, across all farms: *β*-lactams, macrolides, aminoglycosides, and phenicols. Fluoroquinolones (9/10 farms), tetracyclines (7/10), pleuromutilins/fosfomycin (6/10), and sulfonamides/lincosamides (3/10) were also used. Most farms (8/10) used >7 ATM classes (11–18 agents), irrespective of MRSA status (Supplementary Table S3).

### 3.2 Isolation and molecular characterization of MRSA

A farm was considered MRSA-positive if at least one of five collected samples tested positive (Table 1). MRSA was detected in 41/50 samples from 8/10 farms (80%), with 6 farms (IDs 1, 2, 5, 6, 7, 9) having ≥3 positive samples. Finishing (87.5%, 7/8 farms) and nursery (75%, 6/8) categories were most frequently affected. All production categories in farm ID5 tested positive for MRSA (Table 1).

Three clonal complexes (CCs) were identified: (i) CC398-LA-MRSA, detected in five farms (IDs 1, 2, 6, 7, 9) (53.7%, n:22; *sau1-hsdS1*^+^, IEC^¯^, PFGE non-typeable), comprising 17 ST398 isolates [*spa*-t034 (64.7%) and t571 (35.3%); SCC*mec* V (76.4%), V-like (11.8%), III-like (11.8%)] and five ST8814 isolates, a novel single-locus variant (SLV) of ST398 (*gmk*_629) linked to t571/SCC*mec*V; (ii) CC1/ST9-LA-MRSA, found in three farms (IDs 3, 5, 7); [41.5%, n: 17; IEC¯, mainly PFGE-type X3 (94.1%), *spa*-t16964 including an unassigned variant in SA7643 (07–23–02-12-23-12-23-34), SCC*mec*V] and (iii) CC8/ST72-CA-MRSA detected in one farm (ID 4) (4.8%, n:2; IEC+, PFGE-type R2, *spa*-t3092, SCC*mec*IVa). Farm ID7 harbored both LA-MRSA lineages (Tables 1, 2). One representative of each CC398 SCC*mec* variant (SA7624/III-like and SA7628/V-like) was sequenced.

### 3.3 Antimicrobial resistance: phenotypic and genotypic profiles

**Figure 1:**
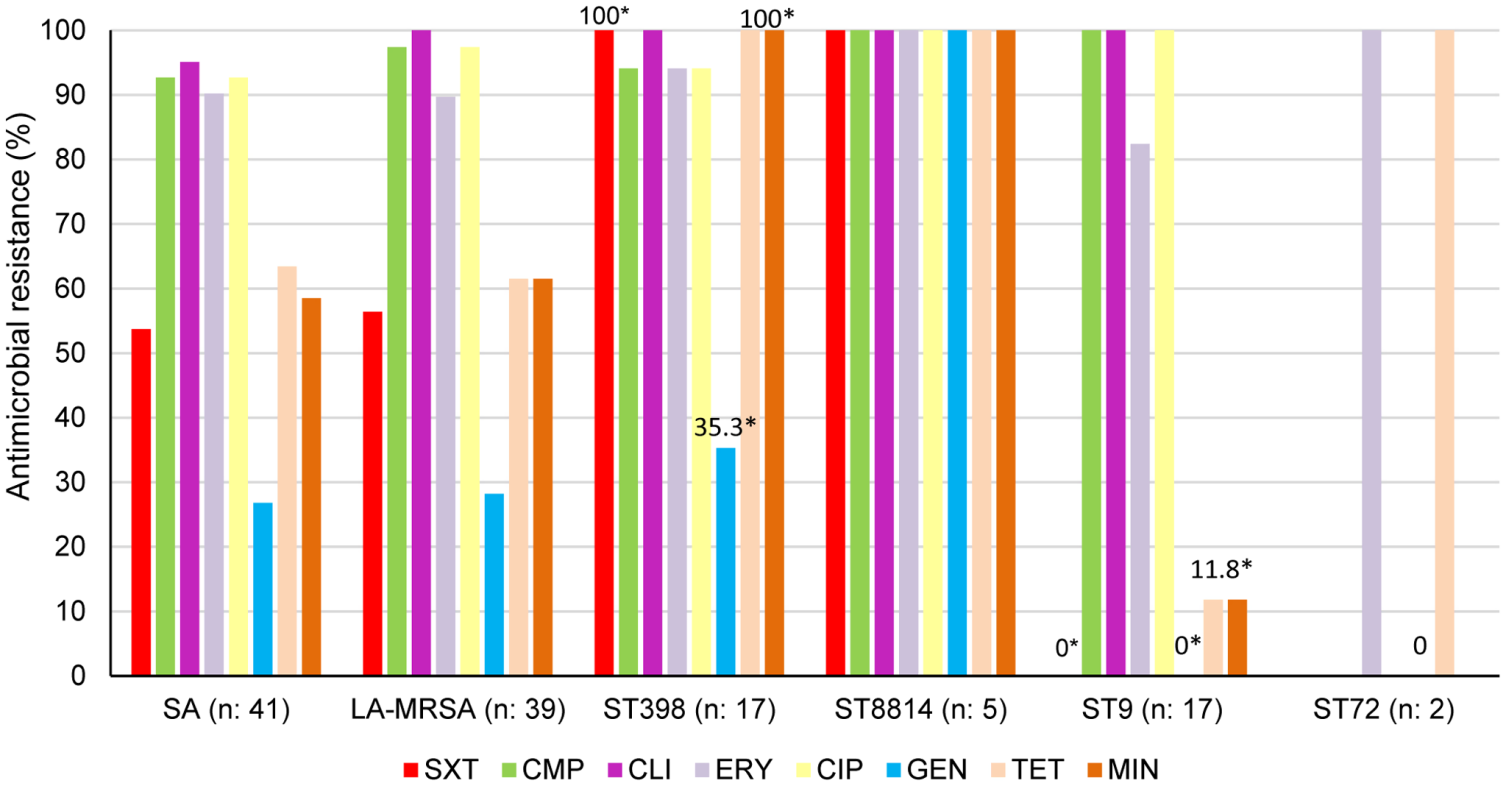
Phenotypic resistance profiles of 41 pig-environment MRSA isolates from the central region of Argentina, including LA-MRSA (CC398 and CC1/ST9), ST398, ST8814, ST9, and ST72, against trimethoprim-sulfamethoxazole (SXT), chloramphenicol (CMP), clindamycin (CLI), erythromycin (ERY), ciprofloxacin (CIP), gentamicin (GEN), tetracycline (TET), and minocycline (MIN). *p < 0.05 by χ^2^ test, comparing MRSA ST398 and MRSA ST9 for resistance to minocycline, trimethoprim-sulfamethoxazole, and gentamicin.

All MRSA isolates (n: 41) were MDR, with resistance to 3–7 drug classes, and grouped into eight resistance profiles. Profiles 1 and 2, both associated with CC398-MRSA, showed resistance to ≥6 classes (Table 2). Resistance to CMP (92.7%), CLI (95.1%, including 85.3% constitutive MLSB, CLI-c and 9.8% lincosamide-only, CLI-l), ERY (90.2%), TET (63.4%), MIN (58.5%), SXT (53.7%), and GEN (26.8%) were detected (Figure 1). No resistance to rifampicin, nitrofurantoin or vancomycin was identified (Supplementary Table S4). Within LA-MRSA, CC398, particularly ST8814, showed significantly higher resistance than CC1/ST9 to MIN (100% vs. 11.8%), SXT (100% vs. 0%), and GEN (35.3% vs. 0%) (*p* < 0.05, Figure 1).

Detected antimicrobial resistance genes included *mecA, tet*(M)/(L)/(K)/(T), *erm*(C)/(T), *msr*(A), *mph*(C), *lnu*(B), *vga*(A), *isa*(E), *fexA, aac(6′)-aph(2″),* and *dfrG*, consistent with phenotypic profiles. The *optr*A gene was found in six isolates (6/41, 14.6%): five ST398 from farm ID1 and one ST9 from ID7 (Table 2). All *optr*A-positive isolates were CMP-resistant with borderline LZD susceptibility (MIC-Vitek2: 2–4 μg/mL; MIC-E-test: 2 μg/mL; Supplementary Table S4). Two were sequenced (ST398: SA7603; ST9: SA7633). *fos*B was detected in 10 ST398 isolates (10/41, 24.3%), from farms ID2 (t571, n:6) and ID6 (t034, n:4); four (two per farm) were sequenced (Tables 1, 2).

### 3.4 Whole-genome sequencing based characteristics

**Figure 2:**
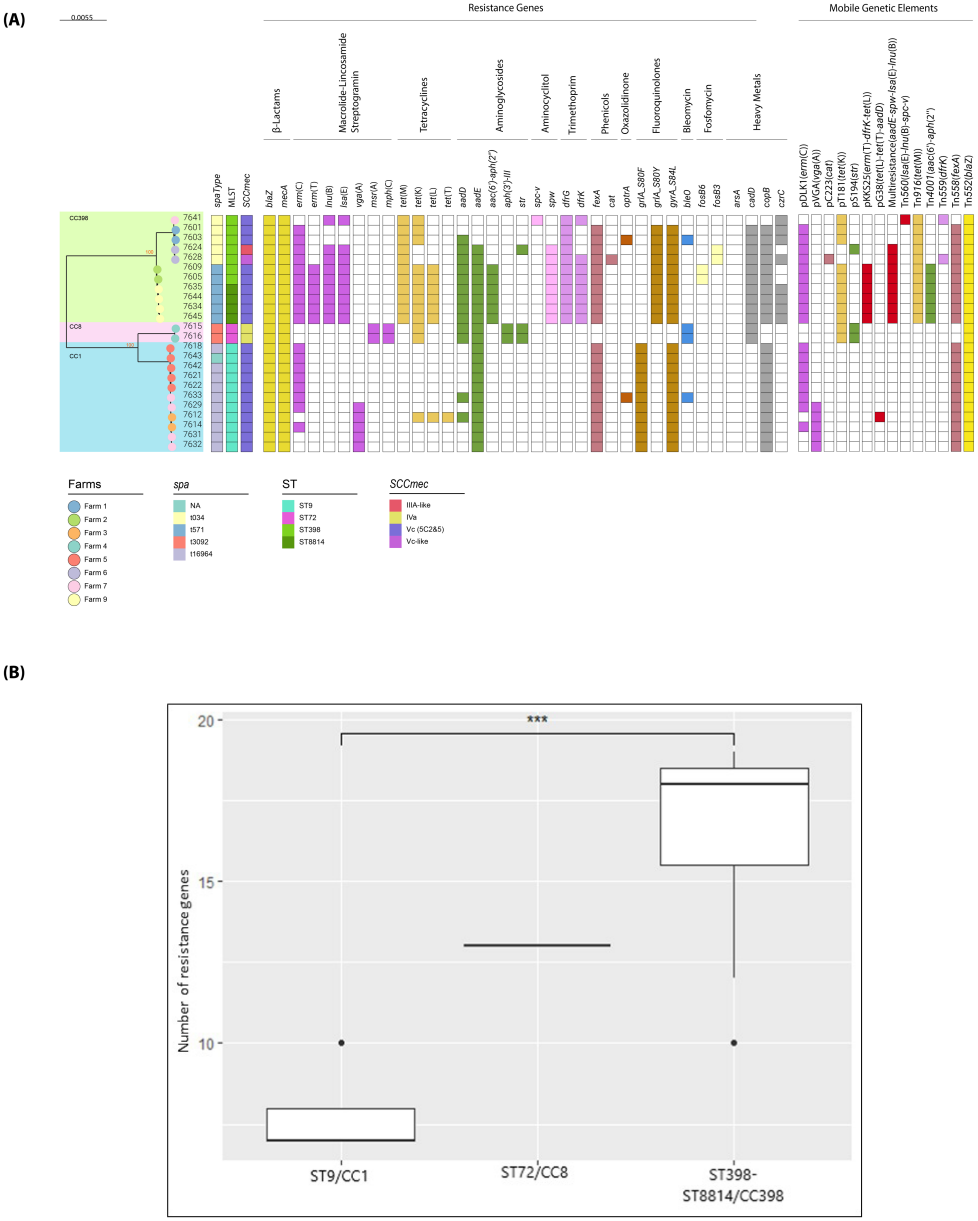
Phylogeny and antimicrobial resistance of Argentine pig-environment MRSA. **(A)** Midpoint-rooted maximum-likelihood phylogenetic tree based on core genome SNPs of 24 MRSA isolates from eight pig farms in central Argentina (Córdoba), constructed with 1,000 bootstrap replicates. *S. aureus* ENA ST425 (strain LGA251; accession FR821779) was used as the reference genome. Farm identifiers (color-coded circles at branch tips), spa type, multilocus sequence type (MLST), SCC*mec* type, and presence/absence of antimicrobial resistance genes (ARGs) and mobile genetic elements (MGEs) are shown. ARGs and *fosB* subtypes were identified using CGE ResFinder (Bortolaia et al., 2020), Pathogenwatch, and the Comprehensive Antibiotic Resistance Database (CARD) (Alcock et al., 2020). MGEs were identified with PlasmidFinder (Carattoli et al., 2014; Carattoli and Hasman, 2020) and MobileElementFinder (Johansson et al., 2021), and mapped/visualized with Proksee (Grant et al., 2023). ARGs are grouped into 11 antimicrobial classes: *β*-lactams, macrolide–lincosamide–streptogramin, tetracyclines, aminoglycosides, aminocyclitols, trimethoprim, phenicols, oxazolidinones, fluoroquinolones, bleomycin, and fosfomycin. Heavy metal resistance genes are also indicated. **(B)** Total number of ARGs per lineage. The bar graph shows ARG counts within each lineage: MRSA ST9/CC1 (n:11), ST72/CC8 (n: 2), and CC398 (n: 11). ***indicates *P* < 0.05 by Tukey’s test.

The maximum-likelihood phylogenetic tree with farm origin, *spa* type, MLST, SCC*mec* type, resistance genes, and major MGEs of 24 sequenced MRSA isolates is shown in Figure 2A and Supplementary Table S5.4. All genomes met quality thresholds (Supplementary Table S2.3). The phylogeny revealed two main clades: CC398, and a second comprising CC1/ST9 and CC8/ST72.

All LA-MRSA carried SCC*mec*Vc (5C2&5), except two ST398 isolates with Vc-like and IIIA-like variants (Supplementary Figures S1, S2A–C). ST72 isolates carried SCC*mec*IVa (Figure 2A). Isolate SA7628 (V-like) had a shortened SCC*mec*Vc lacking *tet*K but included *ccrA1/B1*, resembling strain RD9/(SAMN02391366) (Monecke et al., 2018). Virtual hybridization confirmed presence probes associated with RD9 SCC*mec* [VT + *czrC*+*ccrA1*(*ccrB1*)], including gene D1GU38 linked to a second *ccrC*, supporting a rare Vc variant with an additional *ccrA1B1* complex. The SCC*mec* IIIA-like element in isolate SA7624 lacked the SCCHg region. BLASTn, found the *mecA* complex and *ccrB3/ccrA3* on separate contigs. Virtual hybridization with PCR primers (Chongtrakool et al., 2006) detected probes consistent with SCC*mec*III3A (strain JCSC1716), including *ccrA3B3*-specific primers (*β*3, β4). Its *ccrA3/B3* genes shared 82.7 and 90.1% identity with those of strain 85/2082 SCC*mec*III, respectively. Phylogenetic analysis of known *ccrA* homologs (Huang et al., 2024) placed *ccr*A-7624, located upstream of *ccr*B3, within the *ccr*A3 clade of SCC*mec*III (strain 85/2082), with 99.9% bootstrap support. Although *ccrA*-7624 showed 82.7% identity to *ccrA*3, just below the 85% cutoff for allotype definition, it exceeded inter-allotype ranges [48–81.6% (Huang et al., 2024); 60–78% (Uehara, 2022)], suggesting it belongs to or is closely related to *ccrA3*. Despite lacking IS*431mec* and cLt3 primers (Chongtrakool et al., 2006), results support that SA7624 carries a type III-like SCC*mec* with only the type 3A component.

### 3.5 Phylogenetic analysis and transmission of LA-MRSA

**Figure 3:**
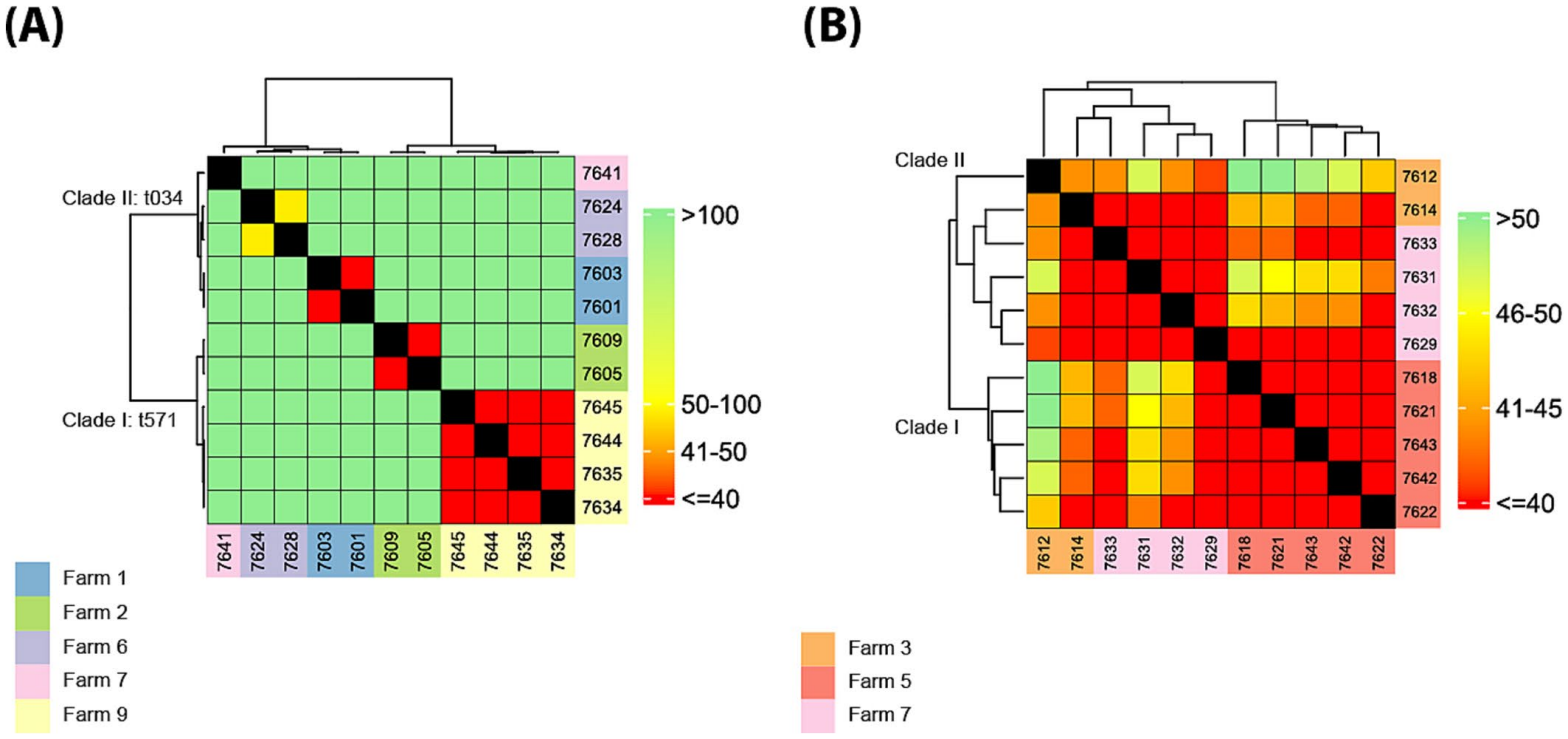
Genetic relatedness of pig–environment MRSA isolates based on core genome SNPs. **(A,B)** Heatmaps and dendrograms showing pairwise SNP distances among 11 CC398 MRSA isolates **(A)** and 11 ST9/CC1 MRSA isolates **(B)** from this study. SNP distances were calculated from core genome alignments, and a threshold of ≤40 SNPs was used to define genetic relatedness or potential transmission clusters. Visualizations were generated using the *ggplot2* (v3.4.4) and *ComplexHeatmap* (v2.15.4) packages in RStudio. Isolates are color-coded by farm of origin.

Core SNP-based phylogenetic trees and pairwise SNP heatmaps assessed relatedness and potential farm transmission (Figures 3A,B; Supplementary Tables S5.1–3), for each major CC. CC398 grouped into *spa*-types *t*571 (Group I) and *t*034 (Group II). Pairwise SNPs in the *t*034 clade (ID1, ID6, ID7) ranged from 9 to 277 (mean: 167.4, SD: 92.3). At ID1, isolates 7,601–7,603 differed by 9 SNPs but were >100 SNPs from ID6 (7,624/7628) and ID7 (7641). ID6 isolates differed by 58 SNPs and harbored distinct SCC*mec* types (V-like vs. III-like), suggesting separate origins. The ID7 isolate was >200 SNPs from all others. In the t571 clade, SNPs ranged from 8 to 232 (mean: 122.7, SD: 100.7). Two ST398 isolates from ID2 differed by 18 SNPs. Four ST8814 isolates from ID9 [lactating (2), nursery (1), finishing (1)] showed 8–26 SNP differences (mean: 17.0, SD: 7.1), with a difference of 20 SNPs between the two isolates from lactating piglets, consistent with within-farm transmission. They were >200 SNPs apart from ID2 isolates, indicating no inter-farm spread. CC398 phylogeny supports within-farm but not between-farm transmission.

For CC1/ST9, SNP distances were 13–56 (mean: 37.8, SD: 10.6), indicating close relatedness. Two clades were identified: Clade I (ID5, n: 5) showed 19–32 SNPs (mean: 24.8, SD: 3.8); Clade II (ID7, ID3; n: 6) showed 13–52 SNPs (mean: 34.3, SD: 11.1). Intra-farm distances: 19–32 SNPs (ID5), 13–40 (ID7, including an 18-SNP difference between two isolates from the same category, breeding stock), and 44 (ID3). The maximum SNP distance within same-farm isolates was 44, supporting the ≤40 SNP threshold, as used by Randad et al. (2021), (≤43 SNPs for CC1/ST9). We identified at least four isolates [7,629 and 7,633 (ID7), 7,614 (ID3), and 7,622 (ID5)] that clustered with those from other farms, indicating inter-farm CC1/ST9 MRSA transmission.

### 3.6 Antibiotic resistance genes and genetic context

Besides *mecA* (PBP2a) and *blaZ* (β-lactamase), 26 ARGs were identified across the 24 MRSA genomes. Their presence, distribution within the clonal complexes, inferred genomic locations (chromosomal vs. plasmid), and MGEs (Figure 2A, Supplementary Tables S5.4,5 and Supplementary Figures S3A,B) are shown. These conferred resistance to macrolides-lincosamides-streptograminB [*erm*(C)/(*T*)], macrolides-streptograminB [*msr*(A)], macrolides [*mph*(C)], lincosamides [*lnu*(B)], lincosamides-pleuromutilins-streptograminA [*isa*(E), *vga*(A)], phenicols-oxazolidinones (*optrA*), tetracyclines [*tet*(M)/(K)/(T)/(L)], aminoglycosides [*aadD*, *aadE*, *aac*(6′)-*aph*(2″), *aph*(3′)-III, *str*], aminocyclitol/spectinomycin (*spw*, *spc-v*), folate pathway inhibitors (*dfrG*/*K*), phenicols (*fexA*, *cat*), fosfomycin (*fosB6*/*B3*), and bleomycin (*bleO*). Three chromosomal mutations conferring fluoroquinolone resistance were also detected: *grlA/parC*(S80F, S80Y) and *gyrA*(S84L). Most genes [e.g., *erm*(C)/(T), *lnu*(B), *vga*(A), *lsa*(E), *aadE, aadD, aac(6′)-aph(2″), str, spc-v, spw, dfrK, tet*(M)/(K)/(L)/(T), *cat, fexA*] were associated with MGEs. Four plasmid replicase families were identified: Rep_trans, RepL, Rep_1, and PriCT_1. Replicons were detected in all strains, and more frequent in CC398 (Supplementary Table S5.6).

All strains harbored multiple ARGs, with significant differences in number and distribution across MRSA lineages. CC398 isolates carried significantly more ARGs than ST9 (*p* < 0.001), especially the novel ST398 SLV: ST8814 (Figure 2B). Compared to ST9, CC398 isolates were significantly enriched (*p* ≤ 0.01) in *erm*(T) [54.5%, on pKKS25 with *dfrK* and *tet*(L), only in *spa*-t571 group]; *lnu*(B)-*lsa*(E) (81.8%), *spw* (72.7%), *tet*(M) (100%, Tn*916*), *tet*(K) (81.8%, on pT181 within SCC*mec*Vc), *aadD* (81.8%), *aac(6′)-aph(2″)* (54.5%, plasmid-borne Tn*4001*, in *spa*-t571 group), *dfrG* (100%, chromosomal in *spa*-*t*034, plasmid-borne in *spa*-*t*571 strains), *dfrK* (72.7%, chromosomal-Tn*559* in two *spa*-t034 isolates,pKKS25-plasmid in *spa*-*t*571 group), and *grlA*(S80Y) fluoroquinolone-resistance mutation (90.9%). The *lnu*(B)-*lsa*(E)-*aadE-spw* cluster (>98% identity to SAC2944, SAC2828, and SAC2829, JQ861959, Wendlandt et al., 2013) predicted to be plasmid-borne, was found in all CC398 isolates except SA7641, where a truncated version (lacking *aadE*) was integrated into *radC* via Tn*560* (Supplementary Figure S3B).

*fosB* genes (*fosB3, fosB6*) were detected exclusively in CC398 MRSA. *fosB6* (100% identity to the *S. aureus* wild-type gene, KR870314, Fu et al., 2016) was plasmid-borne and identified in two closely related spa-t571 isolates (effluent/nursery, 18 SNPs apart) from farm ID2, suggesting within-farm transmission, further supported by PCR detection of four additional fosB-positive *spa*-t571 strains from the same farm (Tables 1, 2). *fosB3* (99.5% identity to *E. faecium* gene on plasmid pEfm-HS0661, HQ219726, Xu et al., 2013; Hu et al., 2023) was found in two *spa*-t034 isolates from farm ID6 (58 SNPs apart, >2,000 SNPs from ID2 strains), likely reflecting independent acquisition under fosfomycin pressure (Supplementary Tables S5.1,4). Both genes were predicted to be plasmid-borne, but genetic context could not be resolved due to their location on short contigs (2.5–3.5 kb) lacking additional genes.

ARGs shared across LA-MRSA lineages included the *bla*Z operon, on chromosomal Tn*552* (present in all isolates, with an additional plasmid copy in CC398 *spa*-t571 group), *erm*(C) on a pDLK1-like plasmid, *fex*A on chromosomal Tn*558* (in all LA-MRSA except CC398-SA7641), *optr*A, and the g*yrA*(S84L) mutation. The *optr*A genes from SA7603(ST398) and SA7633(ST9) were 100% identical and shared 99.4% identity with the *E. faecalis* E349 wild-type gene (GenBank: KP399637). Protein alignment (Supplementary Figure S4) revealed a novel variant, OptrA/FDKFP, with five amino acid substitutions (S147F, Y176D, I287K, L471F, T481P), including two unique mutations (S147F, L471F) per nomenclature (Schwarz et al., 2021). The *optr*A gene showed 99.57% identity to plasmid pL14 (CP043725.2), though its full genetic context could not be resolved due to short contigs (2.19 kb) lacking flanking genes. Both isolates also carried plasmid-encoded *bleO* gene and an Inc18-type plasmid on separate contigs.

In ST9 LA-MRSA, lineage-specific elements included *vga*(A) (on pVGA), *tet*(T) [on pG38 with *tet*(L) and *aad*D], and *grlA*(S80F) mutation. The macrolide resistance genes *mph*(C) and *msr*(A), chromosomally co-located, were associated with ST72 MRSA.

### 3.7 Virulence gene repertoire

**Figure 4:**
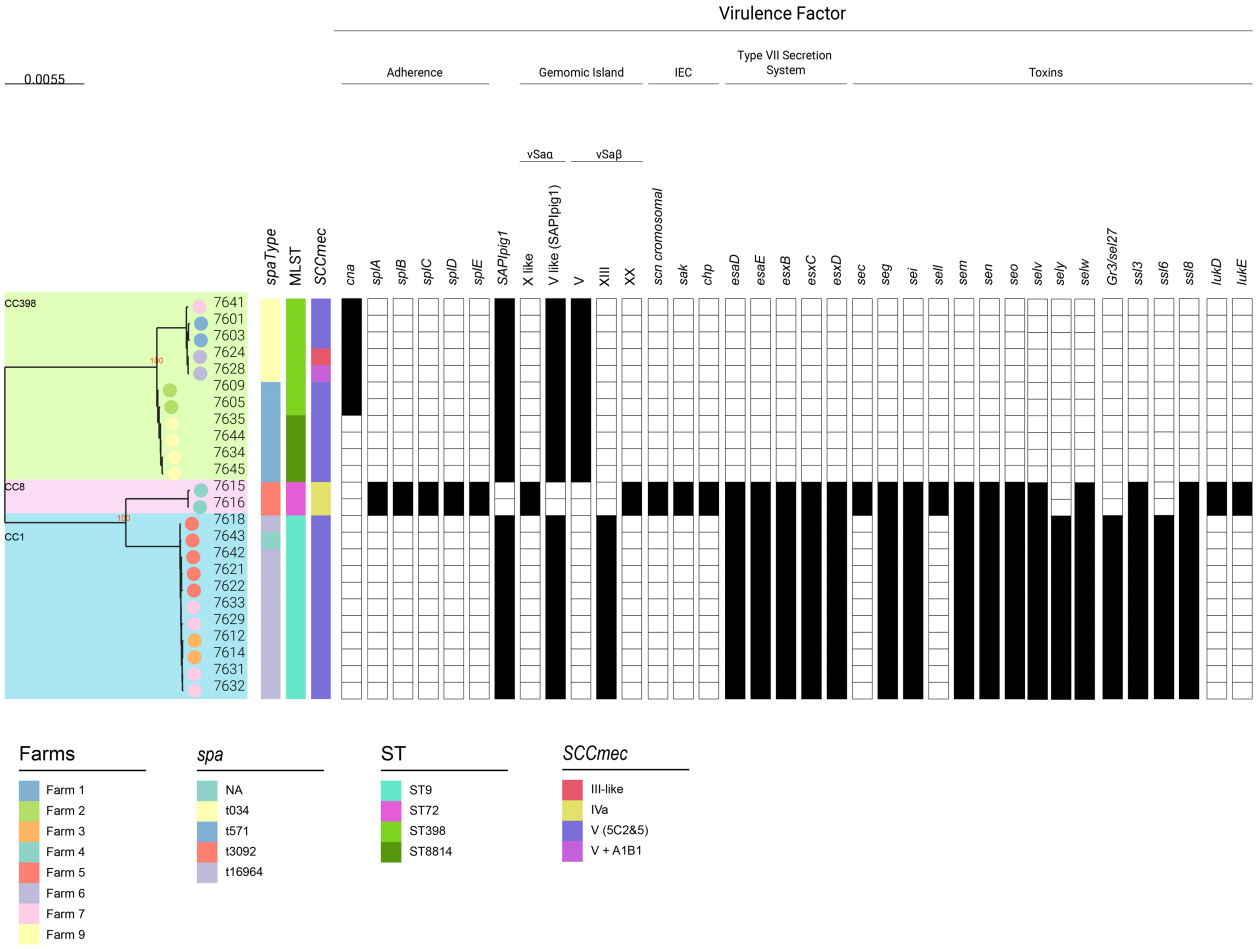
Phylogeny and virulence genes of pig–environment MRSA isolates from Argentina. The phylogenetic tree is the same midpoint-rooted maximum-likelihood tree shown in Figure 2A, constructed from core genome SNPs of 24 MRSA isolates. As in Figure 2A, farm identifiers (color-coded circles at branch tips), spa type, multilocus sequence type (MLST), and SCC*mec* type are indicated. Presence/absence of virulence genes, grouped into functional categories (adherence, immune evasion, toxins, and type VII secretion systems), is displayed. Virulence genes were identified using VFDB (Liu et al., 2022) and VirulenceFinder v2.0 (Joensen et al., 2014), with confirmation by BLASTn. The types of νSaα and νSaβ genomic islands and the presence of SaPIpig1 are also shown. Comparative analyses with reference νSa islands were performed using Mauve v2.4.0, and νSaβ islands were typed based on ≥90% identity (Klaui et al., 2019).

A total of 115 virulence genes were analyzed and grouped into six categories: adherence (n:22), exoenzymes (n:15), immune evasion (n:5), iron uptake (n:6), toxins (n:55), and type VII secretion systems (T7SS; n:12) (Figure 4, Supplementary Table S5.7). Most were widespread, but some were lineage-associated: CC398 isolates carried *cna* (collagen adhesion) gene, whereas CC1/ST9 strains encoded T7SS genes (*esaD/E, esxB/C/D*), superantigen-like genes (*ssl*6/8), and enterotoxin-like genes (*sel*27, *sel*v, *sel*y), with *sel*27 co-localized with the *egc* cluster (*seo, sem, sei, selw, sen, seg*) within the νSaβ type XIII island (GI). *sel*26 and *sel*x were detected across all lineages. Two ST72 CA-MRSA strains uniquely carried *sec-2*, *sell* (νSaα X-like, Supplementary Figure S5), serine proteases (*splA–E*), *lukED* (νSaβ XX with *egc* cluster), and immune-evasion genes (*chp*, *scn*, *sak*). All isolates lacked *sdrF–H*, *splF*, classical enterotoxin genes (*sea, seb, sed, see, seh, sej, sek, sep, seq, selr, selu*), exfoliative toxin genes (*eta–etd*), *pvl* (*lukF/S-PV*), *lukF*-like, *lukM*, and *tsst-1*.

Given their role in *SA* virulence and adaptation, νSaα/νSaβ GIs were analyzed (Figure 4, Supplementary Table S5.7 and Supplementary Figure S5). All ST398 and ST9 carried a type V νSaα island downstream of *guaA*, encoding a SaPIbov4-like element (>99.5% identity to SaPIpig1; MW589252), *ssl1–ssl11*, and *lpl* genes, while ST398 lacked *aadE*, *ssl6*, and *ssl8*, consistent with previous reports (Ji et al., 2021). The ST72 νSaα island structure, identified in several public ST72 genomes, resembled the type X νSaα island of H-EMRSA-15 (Zhou et al., 2018). It shared the SaPImw2-associated flanking genes (*ear, sec-2, sel, int-xis*), while the *ssl* cluster additionally contained *ssl4* and *ssl8*. Therefore, we propose it as a “νSaα variant type X-like” element associated with ST72.

In silico νSaβ analysis confirmed known types (>90% identity): type XIII in ST9 (G19F), type V in ST398 (S0385), and type XX in ST72 (13CEB323STA), showing strong correlation with clonal complexes (Klaui et al., 2019; Schwendimann et al., 2021).

### 3.8 Global phylogenetic analysis of CC398 and ST9/CC1 LA-MRSA isolates from pig farms in Central Argentina

#### 3.8.1 Global phylogenetic reconstruction of CC398 LA-MRSA isolates

**Figure 5:**
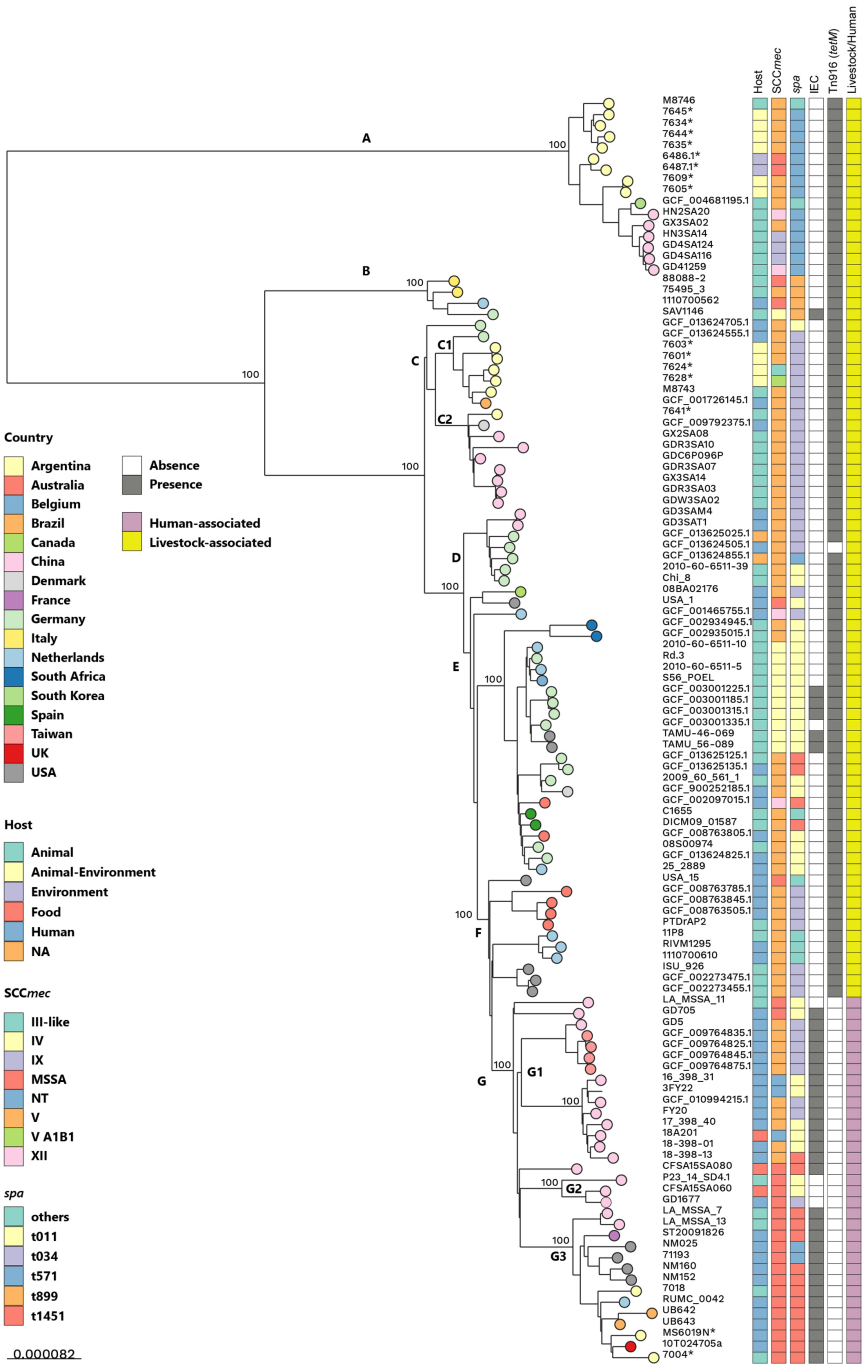
Argentinean pig–environment CC398 MRSA strains in a global context. Midpoint-rooted maximum-likelihood phylogenetic tree based on core genome SNPs from 114 *SA* CC398 genomes, including 16 genomes from central Argentina generated in this study (11 pig–environment MRSA and five MSSA isolates from Córdoba, 2019–2022: two river, two bovine, one human colonization) and 98 representative genomes from public databases that include both livestock- (LA, n: 68) and human-associated (HuA, n:30) clades (Matuszewska et al., 2022) and encompass the major international clades defined by Fernandez et al. (2024). The tree was constructed with 1,000 bootstrap replicates using *S. aureus* S0385/ST398 as the reference genome. Country of origin (color-coded circles at branch tips), host source, SCC*mec* type, *spa* type, MLST, presence/absence of the immune evasion cluster (IEC) and Tn*916* [*tet*(M)], and classification as LA or HuA are indicated. *indicates that the genome was sequenced and analyzed in this study.

The CC398 tree (Figure 5) included 11 pig-environment MRSA and five MSSA genomes from Córdoba (2019–2022; two river, two bovine, one human colonization), analyzed alongside 98 public genomes representing livestock-associated (LA, n:68) and human-associated (HuA, n:30) clades (Matuszewska et al., 2022) and recently defined phylogroups (Fernandez et al., 2024; Supplementary Table S2.1).

Core-SNP analysis confirmed LA clade markers (Tn916^+^, SCC*mec*^+^/MRSA, IEC^−^) (Matuszewska et al., 2022), with pig-environment MRSA and river MSSA isolates clustering within the LA clade (SCC*mec*Vc), while bovine/human MSSA isolates grouped into HuA (IEC^+^, Tn916^−^). Core SNP phylogeny resolved the LA (clades A–F) and HuA (G1–G3) subgroups with 100% bootstrap support, consistent with (Fernandez et al., 2024). LA groups A–F corresponded to clades C6 and C5: group A to C6/American-Asian-phylogroup (AAP), B to C6/European-phylogroup4 (EP4)/L5, C1 to C6/EP4/L1, C2 to C6/EP4/L3, D to C6/EP3, E to C6/EP5, and F to C5/EP2. HuA groups G1–G3 matched clades C3, C1, and C2, respectively.

Argentine CC398 pig-environment MRSA grouped in clades A (spa-t571) and C (spa-t034). Clade A included six MRSA (ID2, n:2; ID9, n:4) and two river MSSA, grouping with C6/AAP reference strains (Fernandez et al., 2024). Four ST8814 isolates from ID9 clustered with a Santa Fe pig isolate (M8746/ST8814), also assigned to C6/AAP, with pairwise SNP distances of 59–98 (mean: 75). River MSSA genomes (SA6486-1, SA6487-1) clustered with ID2 MRSA and pig isolates from China and South Korea. All clade A pig-environment MRSA isolates from Córdoba (*spa*-t571 group) carried SCC*mec*Vc, most (ST8814) with the complete version, along with multiple ARGs mainly associated with MGEs as described in section 4.6.

Clade C comprised five *spa*-t034 MRSA (ID1, ID6, ID7) that grouped with C6/EP4 clade reference strains (Fernandez et al., 2024). Four isolates (ID1 and ID6) belonged to subgroup C1, clustering with a Buenos Aires pig-isolate (M8743; Gagetti et al., 2023b) and a Brazilian human isolate (C6/EP4), showing 96–158 pairwise-SNP (mean: 128) relative to the Argentine pig-environment genomes. A German cattle isolate (C6/EP4/L1) also clustered in C1 (Fernandez et al., 2024). These four isolates shared resistance determinants typical of the EP4/L1 lineage: *aadE–spw–lsa*(E)–*lnu*(B) (2/4), *aadD* (3/4), *tet*(K) (2/4), *dfrG*, *erm*(C), *fexA*, and mutations in *grlA*(S80Y) and *gyrA*(S84L). The fifth isolate (7,641, ID7) clustered in the C2 subgroup with EP4/L3 strains, showing 109–224 SNPs to C6/EP4/L3 isolates from pigs (China) and a Danish. Its resistome included *erm*(C), *spw–lsa*(E)–*lnu*(B) (Tn560), *tet*(K), *dfrG,* and *dfrK* but lacked fluoroquinolone resistance mutations, consistent with EP4/L3 (Fernandez et al., 2024).

Conversely, spa-t1451 MSSA isolates from bovine and human colonization grouped within HuA clade G3, alongside reference strains of clade C2/susceptible phylogroup (C2/SP) (Fernandez et al., 2024), carried *erm*(T), and clustered with predominantly human-origin susceptible strains (Supplementary Table S2.1).

#### 3.8.2 Global phylogenetic reconstruction of ST9/CC1 LA-MRSA isolates

We analyzed 82 SA CC1 genomes (79 ST9, 3 ST109, a SLV of ST9), including 11 pig–environment MRSA, four Córdoba isolates [2019–2022: three human-derived MSSA (one nasal ST9, two infections: ST9 and ST109), one food-derived ST9-MRSA], and 67 public genomes from the Americas, Asia, Europe, and Australia (Supplementary Table S2.2). Public genomes covered ST9 clades defined by Yu et al. (2021), and also included clade C3-MSSA strains from North Carolina (Randad et al., 2021).

**Figure 6:**
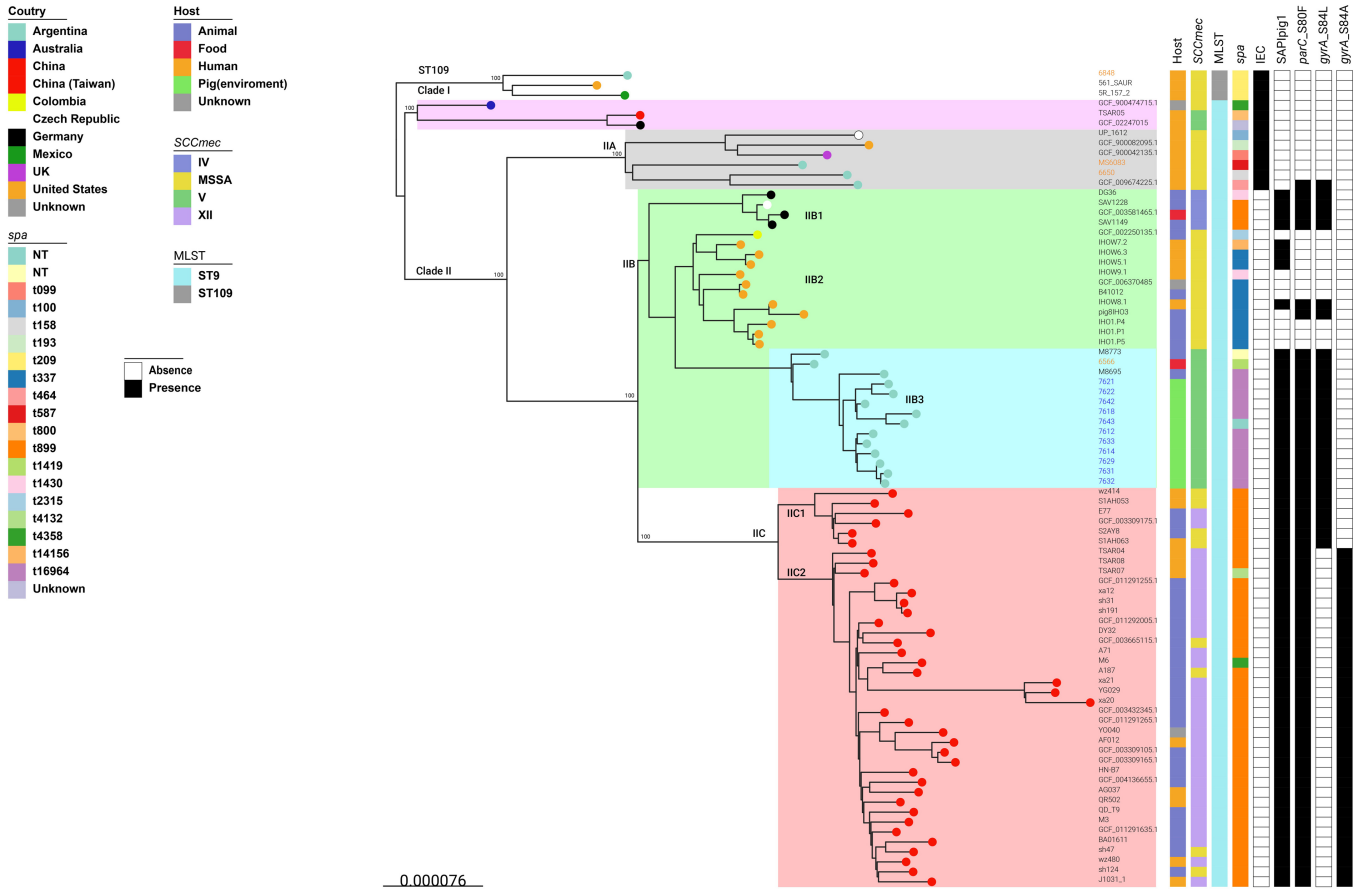
Argentinean pig–environment ST9 MRSA strains in a global context. Midpoint-rooted maximum-likelihood phylogenetic tree based on core genome SNPs from 82 *S. aureus* CC1 genomes, including 79 ST9 and three ST109 (a single-locus variant of ST9). The dataset comprises 15 genomes from central Argentina generated in this study [11 pig–environment MRSA isolates and four additional genomes from Córdoba (2019–2022), including three human-derived MSSA isolates (two ST9 and one ST109) and one food-derived ST9-MRSA isolate], and 67 publicly available genomes representing phylogeographically distinct clades from the Americas, Europe, and Asia. The tree was constructed with 1,000 bootstrap replicates using *S. aureus* QD-CD9/ST9 as the reference genome. Annotations include country of origin (color-coded circles at branch tips), host source, SCC*mec* type, *spa-*type, MLST, presence/absence of the immune evasion cluster (IEC), SaPIpig1, and three chromosomal mutations associated with fluoroquinolone resistance: *parC* (S80F), *gyrA* (S84L), and *gyrA* (S84A). NT (*spa*-type): non-typeable or not assigned by RIDOM (http://spaserver.ridom.de/); unassigned variants shown in different colors.

Core SNP phylogeny (Figure 6; Supplementary Table S2.2) resolved ST9 into two major clades (I and II, 100% bootstrap), distinct from the three ST109 genomes. Clade I comprised three ancestral strains, two Taiwanese MRSA (SCC*mec*V) and one Australian MSSA. Clade II (96.2%, 76/79) was subdivided into IIA, IIB, and IIC clusters, consistent with Yu et al. (2021), supporting phylogenetic robustness. Clade IIA included four reference strains (Yu et al., 2021) and two Cordoba human isolates (SA6650/joint-infection; SA6083/nasal-carrier), all MSSA with a low ARGs content. All clades I and IIA isolates were human-derived (one unknown), IEC^+^, and heterogeneous in *spa-*types.

Clade IIB (n:30; 12 MSSA, 18 MRSA) included all pig-environment MRSA and isolates from the USA, Europe, and Colombia. It formed three subclades (100% bootstrap) with strong regional associations. Subclade IIB1 (n:4): MRSA from European pigs/food; three were clade IIb by Yu et al. (2021). All carried SaPIpig1, *bla*Z, SCC*mec*IV, mutations *gyr*A(S84L)/*grl*A(S80F); most were *spa*-t899. Subclade IIB2 (n:12), MSSA from the USA (n:11; human, pig, unknown) and Colombia (n:1). Most were *spa*-t337, four carried SaPIpig1 and two had triple fluoroquinolone-resistance mutations [*grlA*(S80F)/*grlB*(P585S)/*gyrA*(S84L)]. This group included C3 clade strains (Randad et al., 2021) and three IIb references (Yu et al., 2021). The ARGs distribution across IIB2, IIB3 and IIC clades are shown in Supplementary Table S2.4. Detected ARGs included: *erm*(A), *erm*(C), *lnu*(A), *vga*(A), *tet*(K)/(L)/(T), *aadD*, *spc*, *aac(6′)-aph(2″)*. Subclade IIB3 (n: 14): all Argentine MRSA, 11 pig-environment, one chicken-meat isolate, and two pig MRSA from Buenos Aires/San Luis (Gagetti et al., 2023b). All were MDR and carried *blaZ, mecA, fexA*, *aadE*, SaPIpig1, *grl*A(S80F)/*gyr*A(S84L) mutations, and belonged to *spa*-*t*16964 or variants not-assigned. Additional ARGs included (with variable frequencies): *erm*(C), *vga*(A), *tet*(K)/(L)/(T), *aac*(6′)-aph(2″), *aad*D, *optr*A. Compared to IIB2, IIB3 had higher prevalence of *mecA*, *erm(C)*, *aadE*, *fexA*, and fluoroquinolone-resistance *grl*A(S80F)/*gyr*A(S84L) mutations (*p* ≤ 0.001).

Clade IIC (n.40, 18 MRSA, and 8 MSSA) comprised Chinese human and animal isolates, including IIc reference strains (Yu et al., 2021). Nearly all were *spa*-t899 MRSA harboring SCC*mec*XII and SaPIpig1 and exhibited the highest ARGs burden.

## 4 Discussion

Intensified livestock production and extensive antimicrobial use raise concerns about farm animals as reservoirs of antibiotic-resistant pathogens transmissible to humans (Rhouma et al., 2022; Kasela et al., 2023; Campana-Burguet et al., 2025). In this study, 80% of pig farms in central Argentina were MRSA-positive during February–March 2022, particularly nursery and finishing units, with co-occurrence of major LA-MRSA lineages CC398 and CC1/ST9. All farms had used multiple antimicrobials in the preceding 6 months, underscoring the widespread antimicrobial pressure in these systems. Although our survey covered only 6% of all farms with more than 100 sows in Córdoba (n: 166) (SAGyP, 2023), Argentina’s leading pig-producing province, the high MRSA prevalence aligns with reports from some European countries (Denmark, Netherland, Germany) where LA-MRSA rates reach 70–80%, and contrasts with lower prevalence in Italy (38%), Canada (45%), Sri Lanka (10%), and Japan (8.3–29.6%) (Sieber et al., 2018; Kalupahana et al., 2019; EFSA and ECDC, 2023; Khairullah et al., 2023; Kruger-Haker et al., 2023; Kawanishi et al., 2024). Consistent with previous studies, the fattening and nursery stages were most frequently MRSA-positive, likely driven by increased animal movements and suboptimal hygiene and disinfection practices facilitating bacterial spread (Crombe et al., 2013; Khairullah et al., 2023). A prior Argentine study also reported substantial nasal colonization (29.7%) in fattening pigs (Gagetti et al., 2023b), supporting our findings.

The detection of MDR MRSA in 80% of farms underscores its zoonotic potential, particularly for farm workers with daily animal contact, who may be exposed occupationally while also serving as carriers and vectors to their households and communities (Xin et al., 2022; Kasela et al., 2023; Wang et al., 2023). This risk is amplified by the environmental persistence of LA-MRSA in dust, manure, air, equipment, and improperly handled carcasses, which can also affect wildlife (Abdullahi et al., 2021), facilitating spread beyond farm boundaries and raising concerns for community and food-chain exposure (Xin et al., 2022; EFSA and ECDC, 2023). These findings, consistent with Argentina’s 2022 legislation promoting prudent antimicrobial use (enacted shortly after this study and providing a baseline for future evaluation), also reveal major surveillance gaps in livestock reservoirs, particularly pig farms, recognized hotspots for AMR transmission (Xin et al., 2022), where systematic monitoring is still lacking. Strengthening LA-MRSA surveillance within a One Health framework is therefore critical, encompassing antimicrobial stewardship to reduce selection pressure, enhanced farm biosecurity, including proper management of effluents and carcasses to minimize environmental contamination, regulation of animal movements, and occupational protection for workers (Wang et al., 2023; Khairullah et al., 2023; Sawodny et al., 2025). Together, these considerations highlight the broader public health importance of our findings and underscore the need for coordinated One Health strategies across the animal–human–environment interface to mitigate zoonotic risk and curb AMR dissemination.

LA-MRSA strains in pigs show regional variation: CC398 predominates in Europe, the Americas, Australia, and recently Asia, whereas ST9/CC1 dominates in Asia (particularly China) and occurs sporadically elsewhere, also emerging as LA-MSSA in North America (Randad et al., 2021; Yu et al., 2021; Li et al., 2022; Narongpun et al., 2023; Khairullah et al., 2023; Kawanishi et al., 2024; Campana-Burguet et al., 2025). Although less studied and less widespread than CC398, ST9 has also been implicated in human infections, including cases without direct animal contact (Schwarz et al., 2018; Chen et al., 2020; Avbersek et al., 2021; Yu et al., 2021; Chen et al., 2023). In central Argentina, however, both IEC¯ LA-MRSA lineages were identified: CC398 (*spa*-t034, *spa*-t571; ST398 and the novel ST8814) in five farms, and CC1/ST9 (*spa*-t16964) in three farms, co-occurring in one farm. The unusual coexistence of both globally dominant LA-MRSA clones raises public health concerns due to their potential for interspecies transmission and resistance gene exchange, highlighting the need for molecular surveillance to track lineage evolution, AMR dissemination, and zoonotic risk, which is essential for timely One Health interventions.

In line with previous studies (Richardson et al., 2018), phylogenetic analysis of MRSA isolates revealed lineage-based clustering: CC398 formed a distinct clade, while CC1/ST9 and CC8/ST72 grouped separately. Furthermore, the transmission analysis with core SNP showed intra-farm dissemination of ST398 and ST9 across pig-associated environments in Argentina’s central region, which accounts for 24% of national pig production. While both clones spread within farms, likely between pens or animal categories, only ST9 exhibited inter-farm transmission, highlighting the pig-environment as a reservoir for LA-MRSA, consistent with previous reports (Avbersek et al., 2021; Randad et al., 2021; Li et al., 2022; Li et al., 2022; Hu et al., 2023; Talim et al., 2024). LA-MRSA contaminates soil, water, air, and crops, mainly via manure and airborne particles, facilitating long-distance spread (Wang et al., 2023; Xin et al., 2022). Inter-farm transmission may be driven by animal movements, environmental contamination (e.g., feed, air, water, equipment), human mobility (e.g., farm-workers, veterinarians), and lineage-specific adaptability (Crombe et al., 2013; Kruger-Haker et al., 2022; Chen et al., 2023; Kasela et al., 2023). The restriction of inter-farm spread to ST9 may reflect its greater environmental or host adaptability. Understanding these lineage-specific dynamics is crucial for designing targeted interventions at the animal–environment–human interface, and larger-scale studies including molecular analysis of MRSA are needed to test this hypothesis.

Notably, CC398 exhibits high diversity in *spa* types, STs, and SCC*mec* elements, reflecting ongoing microevolution and evolutionary plasticity (Monecke et al., 2018; Kruger-Haker et al., 2023; Fernandez et al., 2024) as evidenced by the novel ST8814 identified in this study. Consistent with previous findings in pig-associated LA-MRSA (Avbersek et al., 2021; Matuszewska et al., 2022), WGS revealed that most LA-MRSA isolates (CC398 and ST9) carried SCC*mec*Vc (5C2&5), particularly the complete form in CC398MRSA, typically encoding *mec*A, *tet*(K), and *czrC*, conferring resistance to *β*-lactams, tetracycline, and zinc (a common feed additive), thereby supporting the role of co-selection in maintaining resistant strains (Matuszewska et al., 2022; Rhouma et al., 2022; Khairullah et al., 2023). We also detected rare SCC*mec* variants: a Vc-like type with an additional *ccrA1B1* complex and an IIIA-like type containing only the 3A component. Such variants, sporadically reported in CC398 from livestock or clinical isolates, underscore the remarkable plasticity of SCC*mec* (Nemeghaire et al., 2014; Peeters et al., 2015; Monecke et al., 2018; Kruger-Haker et al., 2023; Narongpun et al., 2023; Fernandez et al., 2024). Importantly, SCC*mec* excision and potential horizontal transfer raise concerns about dissemination to more virulent or clinically relevant *S. aureus* lineages, representing an emerging public health threat (Huber et al., 2022; Monecke et al., 2018). Continued surveillance and research are essential to elucidate the selective pressures driving SCC*mec* diversification and its implications for LA-MRSA control.

Consistent with previous reports (Khairullah et al., 2023; EFSA and ECDC, 2023; Wang et al., 2023), we detected a community-associated MRSA lineage (CC8/ST72-t3092-SCC*mec*IVa, IEC^+^) in one farm. Sporadic detection of CA- and HA-MRSA clones primarily associated with humans (e.g., ST5, ST8, ST22, ST30, ST45) in pig-associated environments likely reflects human-to-animal transmission, with potential subsequent host adaptation through MGEs, mutations, or recombination events (Richardson et al., 2018; Matuszewska et al., 2022). Thus, MRSA distribution in pigs is shaped not only by livestock factors but also by regional human clones (Kasela et al., 2023; Khairullah et al., 2023), as illustrated by ST93 dominating in Australia (Sahibzada et al., 2017), USA100 in Canada (Narvaez-Bravo et al., 2016), and an animal-adapted ST5-IV-IEC¯ variant in Japan (Kaku et al., 2022; Kawanishi et al., 2024). In our study, ST72/CC8 was detected on one farm; although still minor in Argentina (Barcudi et al., 2024), it is increasing in Chile (Martinez et al., 2023) and is the predominant CA-MRSA in South Korea, where it circulates among humans, pigs, and the environment (Moon et al., 2019). WGS revealed a novel νSaα variant (type X-like) in ST72 that, similar to other MGEs carrying ARGs and virulence genes in SA (Ba et al., 2023), may facilitate persistence across hosts and environments (Moon et al., 2016). These findings underscore the need for genomic surveillance to monitor the adaptation and potential establishment of MRSA ST72/CC8 in pig farms.

LA-MRSA strains from pigs show resistance to multiple antibiotics commonly used in animal production and human medicine, primarily mediated by MGEs (Matuszewska et al., 2022; Khairullah et al., 2023; Wang et al., 2023; Talim et al., 2024). In this study, all pig-environment MRSA isolates displayed MDR phenotypes, with LA-MRSA showing the broadest profiles, spanning 4–7 antimicrobial classes; over 50% were resistant to six or more, including β-lactams, tetracyclines, phenicols, fluoroquinolones, macrolide-lincosamides, trimethoprim/sulfamethoxazole, and aminoglycosides. In contrast, the two CA-MRSA ST72/CC8 strains exhibited narrower resistance spectra, although broader than those of CA-MRSA lineages circulating in humans in Argentina (Barcudi et al., 2024). Extensive antimicrobial use on these farms and prolonged environmental persistence of some of these, particularly tetracyclines, likely drove the emergence and spread of MDR strains (Rhouma et al., 2022). This represents a significant public health concern, as pig-to-human transmission of LA-MRSA, with broader resistance profiles than HA- or CA-MRSA in Argentina (Barcudi et al., 2024), could further limit treatment options.

Resistance profiles aligned with characteristic ARG patterns of pig-derived SA (Kruger-Haker et al., 2023; Narongpun et al., 2023; Talim et al., 2024; Randad et al., 2021; Ji et al., 2021; Li et al., 2022). These included both commonly reported ARGs in animal-associated staphylococci (e.g., *tet*, *erm*, *blaZ*, *mecA*) and less common or novel determinants such as those conferring resistance to aminocyclitols (*spc-v*, *spw*), fosfomycin (*fosB*), lincosamides–pleuromutilins–streptogramin A [*vga*(A), *lsa*(E)], trimethoprim (*dfrK*), and phenicols–oxazolidinones (*optrA*). Many were linked to MGEs, primarily transposons, small plasmids, or the *spw*-cluster of probable enterococcal origin (Schwarz et al., 2018; Fessler et al., 2018c; Ji et al., 2022; Kruger-Haker et al., 2023). Co-occurrence of multiple ARGs conferring resistance to the same antimicrobial class was frequent, likely due to their co-localization on MGEs (Schwarz et al., 2018; Kruger-Haker et al., 2023). In addition to *mecA* and *blaZ*, shared resistance elements across lineages included *erm*(C), *fexA*, *optrA*, and the *gyrA*(S84L) mutation, all previously reported in LA-MRSA (Yu et al., 2021; Chen et al., 2023; Fernandez et al., 2024). The plasmid-borne *erm*(C), the most common MLS_B gene in staphylococci, was located on a pDLK1-like RepL-type plasmid in both lineages, supporting horizontal transfer. Such plasmids facilitate *erm*(C) dissemination among staphylococci and other Firmicutes, likely driven by macrolide use (e.g., tylosin) in livestock (Lanza et al., 2015; Fessler et al., 2018a, 2018c). The *fexA* gene, encoding an MFS efflux pump for chloramphenicol/florfenicol, was carried on chromosomal Tn*558*, likely reflecting selective pressure from florfenicol use in food animals (Rhouma et al., 2022).

The *optrA* gene, which confers transferable resistance to phenicols and oxazolidinones, is concerning as it may compromise linezolid, a last-resort antibiotic (Schwarz et al., 2021; Timmermans et al., 2021; Li et al., 2022). In Argentina, *optrA*-mediated resistance was recently reported in clinical *E. faecalis* (Gagetti et al., 2023a). We detected *optrA* in six isolates (five ST398 from ID1, one ST9 from ID7), all resistant to chloramphenicol with borderline linezolid MICs (2–4 μg/mL). WGS revealed a novel *OptrA* variant (*OptrA*/FDKFP_SA7603-7633), not previously described (Morroni et al., 2017; Schwarz et al., 2021), in both lineages, suggesting mobilization via MGEs. Variant-specific mutations appeared to variably influence linezolid MICs, suggesting that resistance may depend not only on *OptrA* but also on additional, unidentified factors (Zhu et al., 2020; Schwarz et al., 2021). Thus, the clinical significance of *optrA* in *S. aureus* with MICs near the susceptibility breakpoint remains uncertain. *optrA* was found on short contigs (99.6% identity to the *E. faecalis* pL14 plasmid), limiting contextual resolution. Notably, only the *optrA*-positive isolates carried the plasmid-borne bleO gene and an Inc18-type plasmid. Similar *optrA* contexts, sometimes co-localized with *cfr*, have been reported on plasmids in *S. sciuri, E. casseliflavus*, and LA-MRSA (ST9, ST398) from humans and animals, particularly in China (Schwarz et al., 2021; Li et al., 2022; Dos Santos et al., 2023). Consequently, our results suggest that *optrA* is MGE-associated in both LA-MRSA lineages studied, though hybrid assemblies are needed for confirmation. Our study provides the first evidence of *optrA*-positive *S. aureus* in Argentina, highlighting pig farms as potential reservoirs. Further research is required to elucidate its clinical relevance, zoonotic and environmental dissemination, genetic context, and detection strategies, ultimately defining its public health impact.

In line with previous studies (Ji et al., 2021), CC398 and ST9 displayed distinct AMR profiles and lineage-specific ARG repertoires and contexts, reflecting intra-lineage homogeneity and differential adaptation to antimicrobial use (Fessler et al., 2018b; Jiang et al., 2019; Narongpun et al., 2023). CC398, particularly the novel ST8814, exhibited broader resistance spectra (6–7 classes), with higher rates to minocycline [*tet*(M)], trimethoprim-sulfamethoxazole (*dfrG*), and gentamicin (*aacA-aphD*), linked to a greater number of plasmid- or transposon-borne ARGs promoting horizontal transfer and co-selection (Fessler et al., 2018a). Enriched ARGs included *erm*(T), *lnu*(B)-*lsa*(E), *tet*(M)/(K), *aadD*, *aac(6′)-aph(2″)*, *spw*, *dfrG/K*, and *grlA*(S80Y), many within MGEs such as the enterococcal-derived *aadE*-*spw*-*lsa*(E)-*lnu*(B) MDR cluster, reported in CC398 and ST9 from pigs and humans in Europe and Asia (Wendlandt et al., 2013; Wendlandt et al., 2014; Yu et al., 2021; Kruger-Haker et al., 2023). This cluster was plasmid-borne in most isolates and chromosomally integrated in one *spa*-t034 strain via Tn*560*, a Tn*554*-family transposon described in Chinese porcine ST398 LA-MRSA (Ji et al., 2022; Kruger-Haker et al., 2023). All *spa*-t571 strains exclusively carried *aac(6′)-aph(2″)* on plasmid-borne Tn*4001*, *erm*(T) on a pKKS25-like plasmid, plasmid-borne *dfrG*, and an additional plasmid-borne *blaZ* operon copy. The pKKS25 plasmid, first identified in a porcine ST398 strain from Germany (Kadlec and Schwarz, 2010), harbors *erm*(T), *dfrK*, and *tet*(L), flanked by ISSau*10*, key to ARGs dissemination. Intensive use of *β*-lactams, tetracyclines, aminoglycosides, and macrolides in local pig production likely drove ARGs selection and MGE-mediated co-selection in CC398, especially in the *spa*-t571 group. Consistently, *fosB* subtypes, common in staphylococci (Fu et al., 2016; Hu et al., 2023), were detected in two farms with prior fosfomycin use: *fosB6* in *spa*-t571 strains closely-related from farm ID2, suggesting within-farm transmission, and *fosB3* in *spa*-t034 strains from farm ID6, likely acquired via plasmid transfer or interspecies exchange with enterococci under selective pressure. Similar findings in Chinese duck farms (Hu et al., 2023) underscore farms, as reservoirs of *fosB*-positive MRSA.

In contrast, the plasmids pVGA/[vga(A)] and pG38-like, and the *grl*A(S80F) fluoroquinolone-resistance mutation, were restricted to ST9 MRSA. pG38-like, carrying *aadD*, *tet*(T), and *tet*(L), confers tetracycline-resistance via ribosomal protection/[*tet*(T)] and efflux/[*tet*(L)], is rare in SA, and likely also reflects selection pressure from intensive tetracycline use (Fessler et al., 2018b; Jiang et al., 2019; Narongpun et al., 2023).

Collectively, these findings support the hypothesis that CC398, likely due to the absence of a type I restriction-modification system in νSaβ, is more prone to acquiring exogenous ARGs, enhancing its adaptability (Ji et al., 2021; Kruger-Haker et al., 2022).

Overall, pig-environment LA-MRSA CC398 and ST9/CC1 isolates showed similar virulence profiles, lacking IEC genes (*sak, chp, scn*), consistent with animal adaptation (Wang et al., 2023). Although they did not carry major virulence factors such as Panton-Valentine leukocidin or toxic-shock-syndrome toxin-1, both harbored genes linked to adhesion, colonization, biofilm formation, and diverse infections. They also contained multiple hemolysins and the SaPIpig1 element, a SaPIbov4 variant within νSaα type V, encoding *vwb* (von Willebrand factor-binding protein), which promotes plasma clotting in pigs, sheep, and cattle, enhancing colonization in pigs and its transmission between individuals (Ji et al., 2021). SaPIpig1 also carries *scn* (staphylococcal-complement-inhibitor), potentially facilitating human colonization (Fernandez et al., 2024), underscoring its role in host adaptation and dissemination of ST398 and ST9 (Ji et al., 2021; Fernandez et al., 2024). Lineage-specific virulence traits were identified: CC1/ST9 strains carried additional *sel* genes (*sel27* within νSaβ type XIII with the *egc* cluster, as well as *selv* and *sely*), which together with MDR may increase pathogenic potential and foodborne risk (Schwendimann et al., 2021; Klaui et al., 2019). In contrast, CC398 strains harbored *cna*, encoding a collagen-binding protein linked to osteomyelitis and arthritis (Madani et al., 2017), and a *hysA* homolog within νSaβ type V, previously detected in a human LA-MRSA ST398 endocarditis case and associated with greater virulence than CC1/ST9 (Ji et al., 2021). Collectively, these features highlight the invasive potential of pig-associated LA-MRSA and their public health threat, particularly for immunocompromised individuals or those with comorbidities (Ji et al., 2021; Li et al., 2022; Kasela et al., 2023).

The whole-genome phylogenetic analysis of pig-environment CC398 isolates from Argentina, placed in a global framework, revealed their distribution across two major clades: A (*spa*-t571) and C (*spa*-t034). Both belonged to the LA clade and clustered with C6 clade reference strains (Fernandez et al., 2024), a group driving CC398 dissemination in Europe through the European MRSA phylogroups EP3, EP4, and EP5, and also reported in Asia and the Americas as part of the American–Asian phylogroup (AAP). Within clade A (*spa*-t571), MRSA from Córdoba (farms ID2, ID9), including the novel ST8814, clustered with MSSA strains from a local river and previously identified C6/AAP strains (Fernandez et al., 2024). This group also included a pig isolate from Santa Fe province (Gagetti et al., 2023b) and strains from China and South Korea (Li et al., 2022). These *spa*-t571 MRSA formed a distinct lineage, separated by over 3,600 SNPs from other clades, consistent with earlier studies (Li et al., 2022; Fernandez et al., 2024). The AAP phylogroup, predominantly *spa*-t571, includes both MSSA and MRSA primarily from pigs and humans in Asia and the Americas. These strains lack IEC and *pvl* but carry multiple MGE-associated ARGs, enhancing bloodstream survival, host adaptation, and dissemination (Fernandez et al., 2024; Ba et al., 2023). The ARG profile of Argentina *spa*-t571 isolates aligned with that of the AAP phylogroup, including Tn*558* carrying the florfenicol-chloramphenicol exporter (*fex*A), predominantly found in AAP strains (Fernandez et al., 2024). While most AAP strains harbor SaPIpig2 within νSaα, one U. S. MSSA isolate carries SaPIpig1 (Fernandez et al., 2024), both variants of SaPIbov4. Strikingly, all Argentine CC398 isolates carried SaPIpig1, underscoring complex evolutionary dynamics and local adaptation.

Five pig-environment MRSA *spa*-t034 isolates from Córdoba clustered within clade C, specifically with C6/EP4 phylogroup representative strains (Fernandez et al., 2024). EP4 comprises Danish pig lineages L1–L4 and the Italian L5 (*spa*-t899), which have spread beyond their original region and exhibit diverse ARG profiles. Four isolates (farms ID6, ID1) grouped within subgroup C1, alongside a Buenos Aires pig isolate (Gagetti et al., 2023b), a Brazilian human LA-MRSA, and a German cattle strain (C6/EP4/L1) (Fernandez et al., 2024). These carried L1-typical ARG profiles, including *optr*A and SCC*mec* types Vc, Vc-like A1B1, and IIIA-like. In contrast, one isolate from farm ID7 clustered in subgroup C2 with C6/EP4/L3 strains and carried an ARG profile consistent with L3. Thus, *spa*-t034 Argentine pig-environment MRSA showed close relatedness to EP4 lineages L1 and L3, which include isolates detected in pigs and humans (some IEC^+^) in Denmark, Hungary, and Germany, in pigs from China (L3 only), and also reported in Brazil (Fernandez et al., 2024).

These findings reveal multiple introductions and circulation of international CC398 phylogroups in Argentine pig farms, including C6/AAP and C6/EP4. Local isolates cluster with strains from Asia, Europe, and the Americas, highlighting the global spread of CC398, likely facilitated by animal trade, and the establishment of foreign clones with zoonotic potential. Although Argentina has largely local pig production, live pig imports, mainly from Brazil (91.1% in 2022) (SAGyP, 2023), may have introduced AAP and EP4, both reported in Brazil. Importantly, these results underscore the need to implement or reinforce strategies to prevent LA-MRSA transmission, particularly via animal trade (Sawodny et al., 2025). On the other hand, detection of MSSA CC398 in river water from the same region, assigned to the AAP phylogroup, further suggests environmental reservoirs and possible local MSSA-to-MRSA evolution. Broader SA genomic studies from farm workers, animals, and environments are warranted to explore these dynamics.

Contemporaneous MSSA CC398 isolates from humans and cattle in Córdoba clustered within the HuA clade (subgroup G3), corresponding to the C2/susceptible phylogroup (C2/SP) described by Fernandez et al. (2024). The subgroup G3 isolates, IEC^+^ and predominantly spa-t571 and spa-t1451, carried *erm*(T) (Fernandez et al., 2024) and shared features with Argentina’s main MSSA clone (MSSA398-*erm*(T)-*spa*-t1451) (Di Gregorio et al., 2023; Barcudi et al., 2024). The C2/SP phylogroup is widespread in human colonization and infections across China, Europe, and the Americas, often in individuals without livestock contact (Bonnet et al., 2018; Wardyn et al., 2018; Fernandez et al., 2024), and has also been reported in cattle, pigs, and cats (Tegegne et al., 2022; Fernandez et al., 2024). Our findings reveal co-circulation of distinct MSSA CC398 lineages, C6/AAP (*spa*-t571) and C2/SP (*spa-*t571, *spa*-t1451), in the same region, each with distinct ARGs profiles and molecular traits. This diversity may reflect differences in host adaptation and transmission, underscoring the need to investigate MSSA CC398 under a One Health framework.

Similar to CC398, diverse SC*Cmec* elements (e.g., types III, IV, V, IX, XI, XII/XII-like, with multiple *ccr* complexes, and non-typeable variants) have been reported within CC1/ST9 across studies. Their distribution appears region-specific: ST9 from Europe often carry SCC*mec*IV, while those from China typically harbor SCC*mec*XII (Chuang and Huang, 2015; Jiang et al., 2021; Ji et al., 2021; Yu et al., 2021; Chen et al., 2023).

Global phylogenetic analysis of Argentine ST9/CC1 pig-environment isolates confirmed the strong regional clustering of LA-ST9 lineages (Jiang et al., 2021; Yu et al., 2021; Randad et al., 2021; Ji et al., 2021). Ninety-six percent of ST9 genomes clustered within clade II, comprising human-associated MSSA (IIA, IEC^+^) and livestock-associated clusters IIB and IIC (IEC¯). The two antimicrobial-susceptible ST9 MSSA human isolates from this study grouped within the human-associated IIA clade, while the ST9 MRSA strains from pig environments and food clustered within the LA clade (IIB3). Clade IIB split into three geographically-associated subclades: IIB1 (European MRSA) (Yu et al., 2021), IIB2 (North American MDR-MSSA) (Randad et al., 2021), and IIB3, composed exclusively of Argentine MRSA isolates. Bidirectional pig–human transmission of clades IIB2 and IIC, with potential for community spread, has been reported (Randad et al., 2021; Yu et al., 2021), supporting the zoonotic potential of clade IIB3. IIB3 isolates, carried *spa*-t16964 or related types, SaPIpig1, and exhibited an MDR profile enriched, compared to IIB2, for *erm*(C), *aad*E, *fexA*, and fruorquinolone-resistance mutations *grlA*S80F and *gyrA* S84L. Notably, *fexA* on Tn558, *aadE*, and MRSA (SCC*mec*V) status were exclusive to IIB3, a pattern also observed in the Chinese IIC lineage, which harbors the highest ARG burden (Randad et al., 2021; Yu et al., 2021). The *spa*-t16964 detected here, also reported in MSSA from Germany (Ridom S*pa*Server), differs by one repeat from *spa*-t1430, previously associated with ST9 MSSA and MRSA from pigs, humans, and meat (Yu et al., 2021; Abdullahi et al., 2023).

Host adaptation of *S. aureus* after human-to-animal jumps has been associated with genes or MGEs acquisition and recombination enhancing transmission, colonization, and virulence (Lowder et al., 2009; Richardson et al., 2018; Murray et al., 2017). The clustering of two Argentinean human MSSA ST9 IEC^+^ isolates within clade IIA, which includes IEC^+^ human MSSA of diverse *spa*-types from several countries and corresponds to the ancestral clades described by Yu et al. (2021), supports a human MSSA origin of regional ST9 LA-MRSA clones. This host shift likely involved loss of human-specific virulence factors (IEC), acquisition animal-associated factors such as *vwb* on SaPIpig1 (a SaPIbov4-like element) within the νSaα island, and subsequent antimicrobial-driven evolution through the acquisition of resistance determinants (including SCC*mec* types) across *spa* types. Our data further suggest that IIB2 and IIB3 diverged from a common ancestor, possibly in the Americas, and evolved as independent, geographically segregated clades distinct from the dominant Asian IIC, reflecting region-specific antimicrobial selection (Randad et al., 2021; Yu et al., 2021).

Notably, the co-circulation of two ST9 sublineages in Argentina, non-MDR MSSA in humans and MDR-MRSA in pigs, also demonstrated here, highlights the importance of molecular surveillance to monitor interspecies transmission and ongoing genomic adaptation of this emerging LA-MRSA clone.

Finally, the MDR profile of this regional ST9 LA-MRSA lineage (clade IIB3), combined with its high farm-to-farm transmissibility, detection in ready-to-eat chicken, and co-circulation with international CC398 phylogroups, underscores the need to identify and implement the most effective combination of targeted interventions, including enhanced biosecurity, reduced antimicrobial use, routine pig screening, environmental decontamination, and controlled animal movement, to control LA-MRSA spread in this region and prevent potential zoonotic spillover within a One Health framework (Kasela et al., 2023; Khairullah et al., 2023; Sawodny et al., 2025).

In conclusion, this study reveals a high burden of LA-MRSA in pig farms environments in central Argentina, with 80% of farms testing positive, particularly in nursery and finishing units, during early 2022. Antimicrobial use was widespread, with most farms reporting the use of more than seven drug classes and up to 18 different antimicrobials in the previous 6 months. Molecular characterization identified the co-circulation of two dominant multidrug-resistant lineages: CC398 (ST398 and the novel ST8814) and CC1/ST9, each associated with SCC*mec* Vc variants and pig-adapted resistance profiles. Notably, this is the first report of the phenicol-oxazolidinone resistance gene *optrA* in SA in Argentina, detected in both ST398 and ST9 MRSA isolates. WGS revealed a novel *optrA* variant (*OptrA*/FDKFP_SA7603-7633), likely linked to a MGE, raising concerns about unrecognized reservoirs and potential environmental dissemination. Phylogenetic analysis in a global context showed the emergence of a unique regional ST9 lineage in Argentina, composed exclusively of MRSA from pigs, pig environments, and food, with evidence of intra- and inter-farm transmission. In contrast, CC398 strains clustered within internationally recognized subgroups (C6/AAP and C6/EP4), suggesting multiple introductions and successful local establishment.

This study’s strengths include its prospective design, the comprehensive molecular and genomic characterization of pig–environment MRSA isolates and the identification of a novel *optr*A variant. The integration of recent on-farm antimicrobial use data adds valuable context. Importantly, to our knowledge, this is the first study to estimate the regional prevalence of MRSA-positive pig farms in central Argentina, a major swine-producing area with previously limited data. It also provides insights into LA-MRSA transmission dynamics within and between farms and situates Argentinean pig–environment LA-MRSA lineages within a global phylogenetic framework.

This work has several limitations. First, the small farms sample size (n: 10) limits broader extrapolation. Nonetheless, our findings are supported by a previous study reporting 29.7% nasal MRSA colonization in pigs from farms in three other provinces, with four sequenced strains clustering with those from this study. Second, analyzing only 1–2 MRSA colonies per environmental sample may have underestimated farm-level MRSA diversity and missed co-occurrence of ST398 and ST9. Third, the exclusive use of environmental samples likely led to under detection; including pig nasal samples could have increased the number of positive farms. However, the high proportion of MRSA-positive farms among those sampled highlights the relevance of our findings. Although the detected strains carry genetic features linked to human colonization and infection, no human samples were included to assess zoonotic transmission. Finally, hybrid sequencing will be required to fully characterize the *optrA* genetic context and SCC*mec* variants through complete genome assemblies.

This genomic and molecular analysis of pig–environment MRSA in central Argentina expands current knowledge by highlighting the pig environment as an emerging reservoir of LA-MRSA and ARM. Given their zoonotic potential and capacity for dissemination across farms and human populations, strengthening One Health surveillance and implementing integrated biosecurity and control measures are essential to limit LA-MRSA spread and the AMR dissemination. Addressing MRSA in pig farming is therefore not only an animal health issue but also critical for protecting public health and ecological integrity.

## Data Availability

The datasets presented in this study can be found in online repositories. The names of the repository/repositories and accession number(s) can be found in the article/Supplementary material. Sequence reads of *S. aureus* strains from this study are available from EMBL-EBI Study PRJEB88131.

## Footnotes

^1^ https://www.boletinoficial.gob.ar/detalleAviso/primera/270118/20220824

^2^ www.infostat.com.ar
