# Supplementary material for: Phylogenomic insights into LA-MRSA from Argentine pig farm environments: novel OptrA variant and regional emergence of an ST9 lineage co-circulating with international CC398 lineages
Source: Front Microbiol. 2025 Oct 9;16:1662779. doi: 10.3389/fmicb.2025.1662779 (PMC12557574; doi:10.3389/fmicb.2025.1662779)
Supplement: Supplementary file 1 [file Data_Sheet_1.PDF]

Supplementary Figure 1

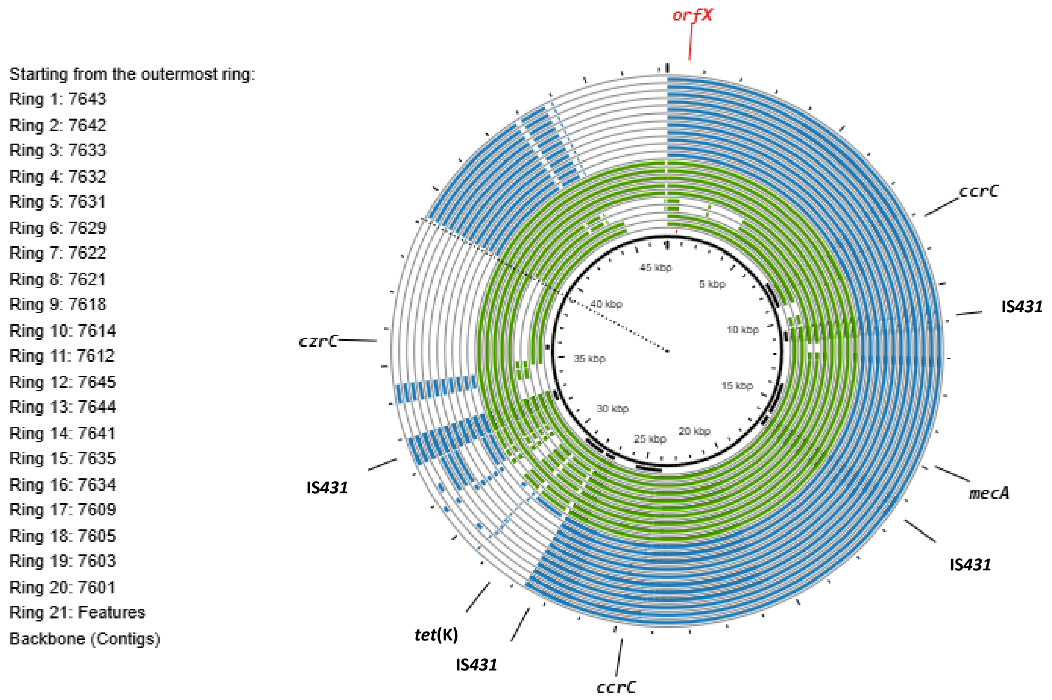

Supplementary Figure 1 Gene map of the type Vc SCC*mec* element in CC398 (green) and ST9 (blue). The map was generated using Proksee, with annotations based on the reference strain *S. aureus* (NCBI accession no. AB505629.1)
