# Supplementary material for: Phylogenomic insights into LA-MRSA from Argentine pig farm environments: novel OptrA variant and regional emergence of an ST9 lineage co-circulating with international CC398 lineages
Source: Front Microbiol. 2025 Oct 9;16:1662779. doi: 10.3389/fmicb.2025.1662779 (PMC12557574; doi:10.3389/fmicb.2025.1662779)
Supplement: Supplementary file 2 [file Data_Sheet_2.PDF]

Supplementary Figure 2

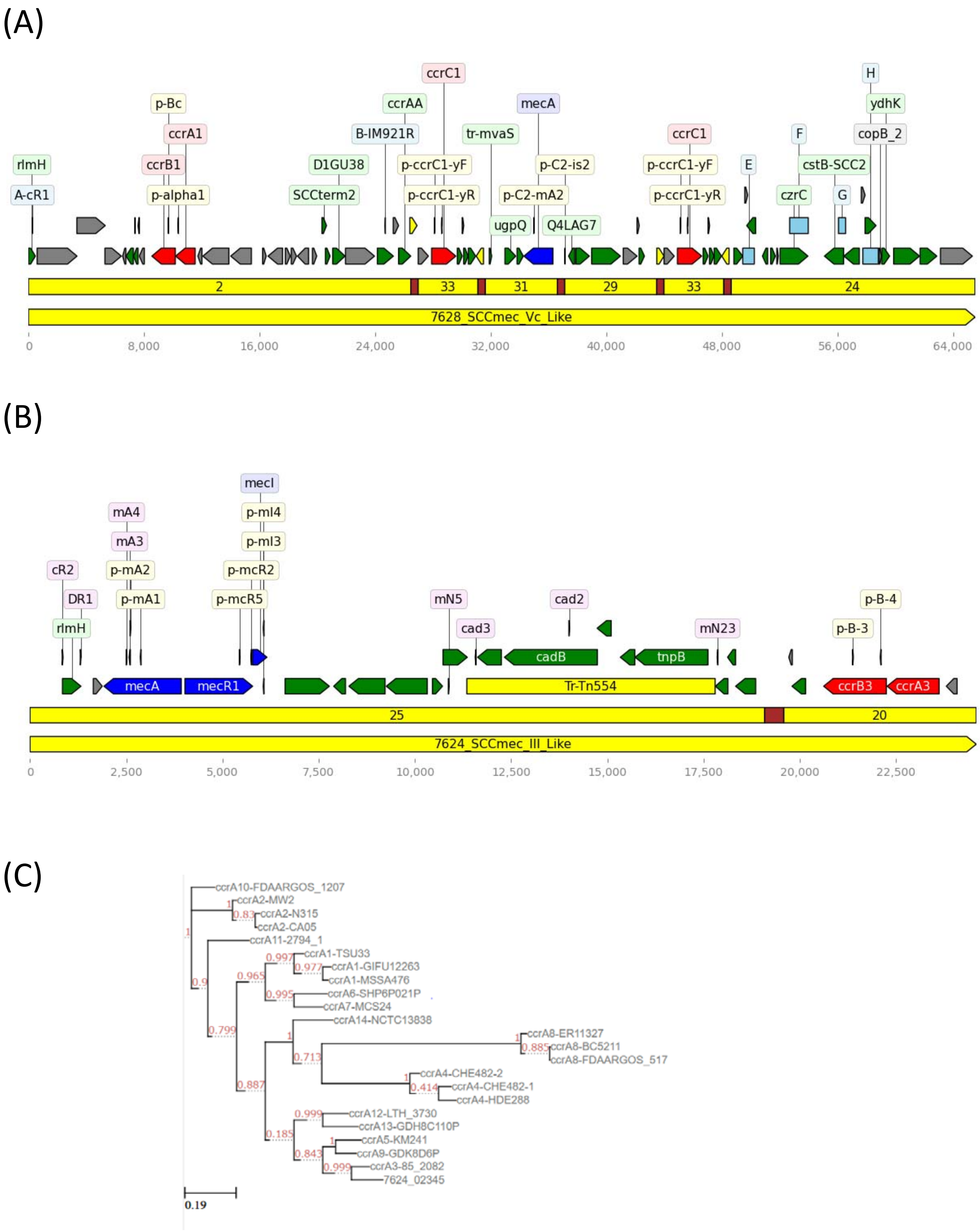

**Supplementary Figure S2. Variant SCC*mec* structures identified in this study. (A, B)** Schematic representations of SCC*mec* variants based on reference nucleotide sequences: JCSC6944 (AB505629.1) for SCC*mec* Vc (A) and 85/2082 (AB037671) for SCCmec IIIA (B). Comparative analyses were performed with Mauve v2.4.0 (Darling et al., 2004; Rissman et al., 2009) and custom scripts. SCC*mec* elements were identified by BLASTn against an extended SCC*mec*Finder database; fragmented elements were resolved using full assemblies and high-identity mapping (>80%, ≥1000 bp). Figures were generated with DNA Features Viewer (https://www.biorxiv.org/content/10.1101/2020.01.09.900589v1). Green arrows indicate regions homologous to the reference genome (>90% identity); gray arrows, regions absent from the reference; red arrows, recombinase genes; blue arrows, the *mecA* gene; yellow arrows, the insertion sequence IS*431*; and dark red blocks, gap regions. SCC*mec* variants were further characterized by virtual hybridization using: (A) array probes to differentiate SCC*mec* variants in CC398-MRSA (Monecke et al., 2018) shown in light green and sequence regions defining structures A–H in the JCSC6944 SCC*mec* element (Li et al., 2011) shown as light blue squares; (B) PCR primers targeting the SCC*mec* IIIA element of strain JCSC1716 (Chongtrakool et al., 2006) marked in violet. In both panels (A) and (B), primer binding sites used for PCR detection (Chongtrakool et al., 2006) are highlighted in light yellow. **(C)** Phylogenetic relationships among 23 *ccrA* homologs, including the *ccrA* gene from strain SA7624. Nucleotide sequences were aligned with MAFFT, and a maximum-likelihood tree was inferred using FastTree with 2,000 bootstrap replicates. Strain names carrying each *ccrA* allele are shown next to the corresponding gene names. The tree was visualized with the ETE Tree online tool.
