# Supplementary material for: Phylogenomic insights into LA-MRSA from Argentine pig farm environments: novel OptrA variant and regional emergence of an ST9 lineage co-circulating with international CC398 lineages
Source: Front Microbiol. 2025 Oct 9;16:1662779. doi: 10.3389/fmicb.2025.1662779 (PMC12557574; doi:10.3389/fmicb.2025.1662779)
Supplement: Supplementary file 3 [file Data_Sheet_3.PDF]

Supplementary Figure 3

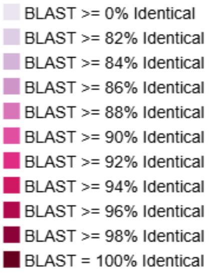

(A) Schematic representation of small plasmids

pDLK1-like/NC_019139.1 **(n:18)**

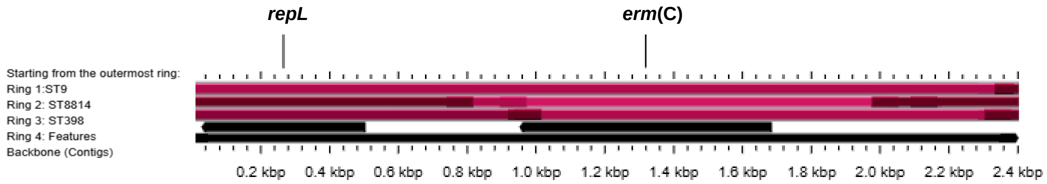

pVGA-like/FJ207465.1 **(n:5)**

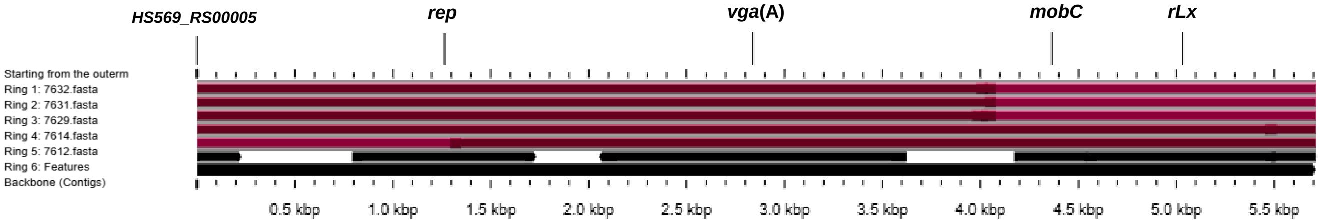

pC223-like/NC_005243.1 **(n:1)**

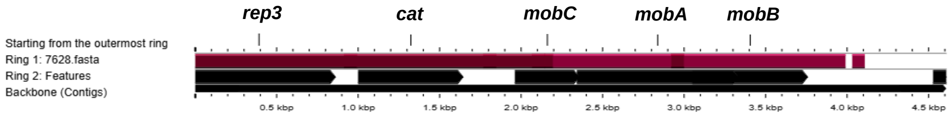

pT181-like/NC_001393.1 **(n:11)**

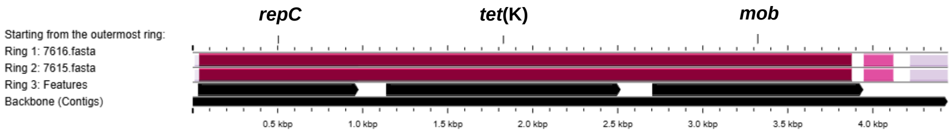

pS194-like/ NC_005564.1 **(n:3)**

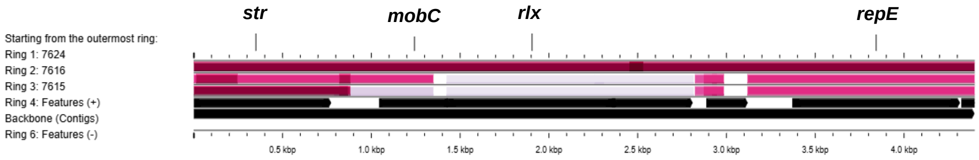

pKKS25-like/ FN390947.**1 (n:6)**

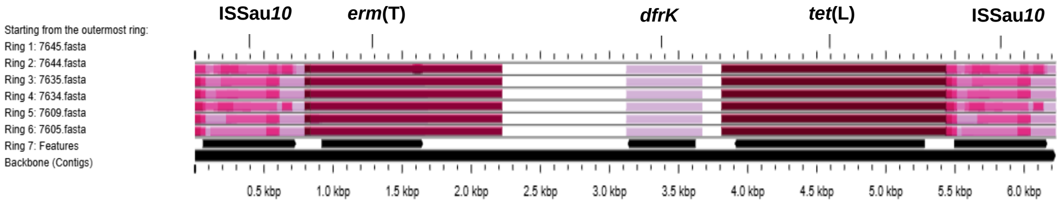

pG38-like/RCDF01000030.1 **(n:1)**

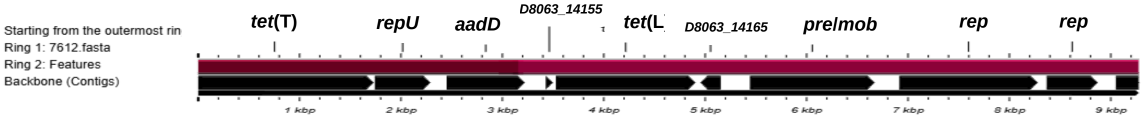

(B) Schematic representation of small transposons and the *spw* cluster

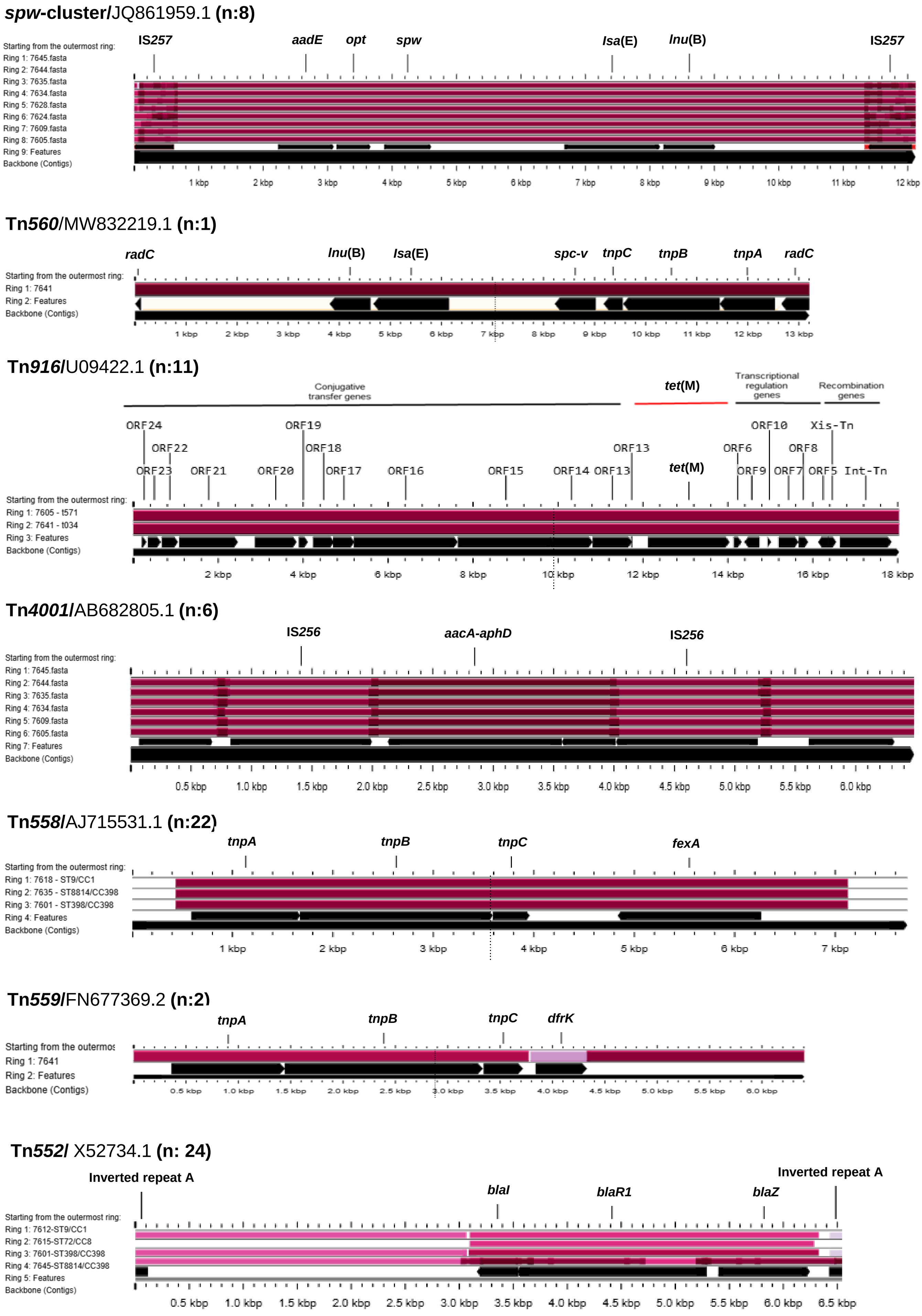

Supplementary Figure 3 . Schematic representation of small plasmids, transposons, and the *spw* gene cluster in pig–environment LA-MRSA lineages.

(A, B) Schematic comparison of (A) small plasmids and (B) small transposons, including the *spw* cluster, identified in CC398 and ST9/CC1 LA-MRSA isolates from pig environments in central Argentina. Maps were generated using Proksee (Grant et al., 2023), with annotations based on reference sequences (accession numbers indicated after each plasmid/transposon name). The number of isolates carrying each mobile genetic element (MGE) is shown in parentheses. Open reading frames (ORFs) from the reference sequences are depicted as arrows, with arrowheads indicating transcriptional orientation. Sequence identity (%) of the study isolates relative to the references is color-coded
