# Supplementary material for: Phylogenomic insights into LA-MRSA from Argentine pig farm environments: novel OptrA variant and regional emergence of an ST9 lineage co-circulating with international CC398 lineages
Source: Front Microbiol. 2025 Oct 9;16:1662779. doi: 10.3389/fmicb.2025.1662779 (PMC12557574; doi:10.3389/fmicb.2025.1662779)
Supplement: Supplementary file 4 [file Data_Sheet_4.PDF]

Supplementary Figure 4

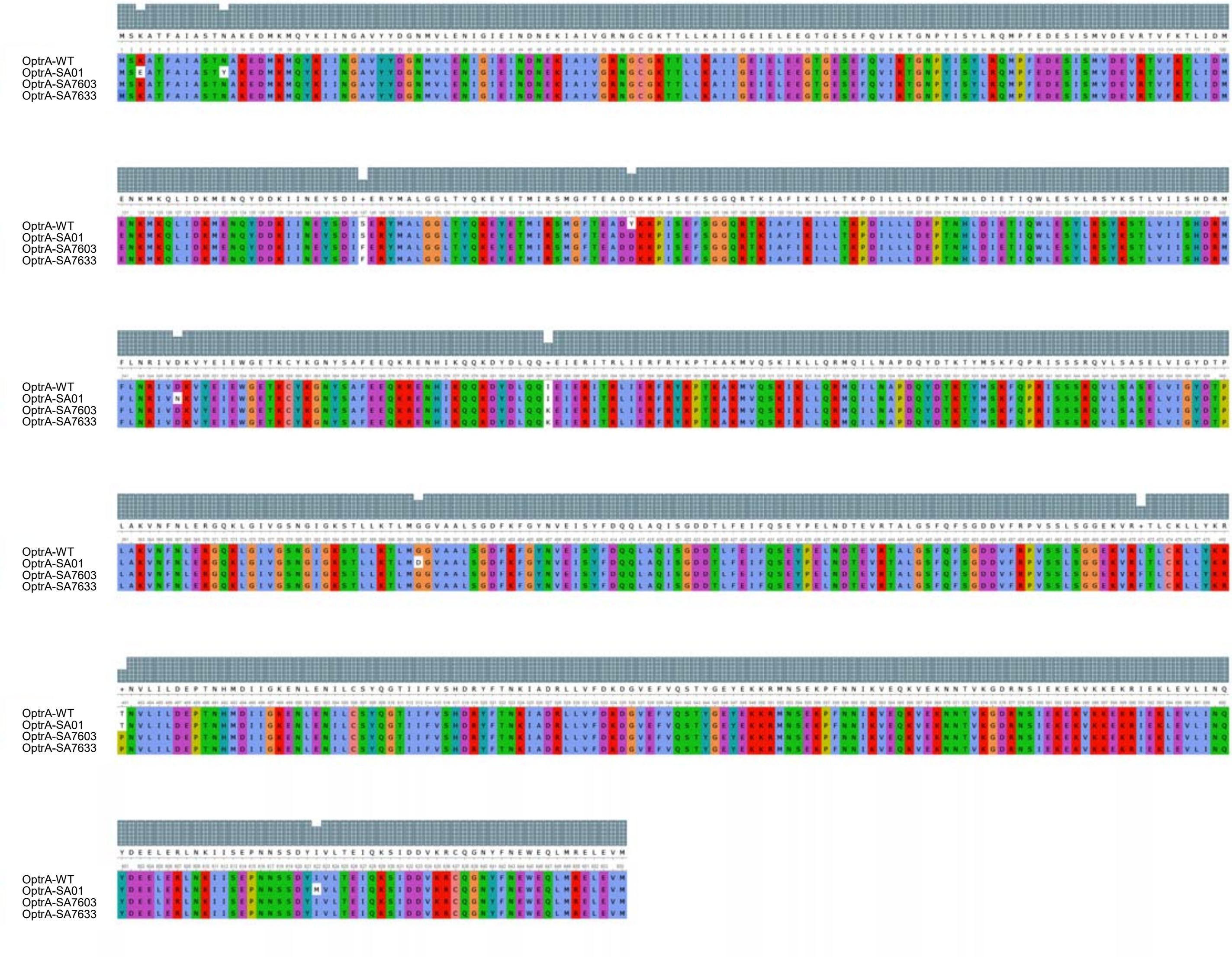

Supplementary Figure 4. Multiple alignment of OptrA amino acid sequences. Amino acid sequences of the OptrA protein, deduced from the *optrA* gene of pig–environment MRSA isolates SA7603 and SA7633, and *S. aureus* SA01 (WP_159314661.1, EYDNDM variant), were aligned against the wild-type OptrA reference protein (WP_063854496.1). Amino acids are color-coded by residue type; substitutions relative to the reference are indicated with a white background. OptrA variants were defined as per Schwarz et al. (2021)
