# Supplementary material for: Phylogenomic insights into LA-MRSA from Argentine pig farm environments: novel OptrA variant and regional emergence of an ST9 lineage co-circulating with international CC398 lineages
Source: Front Microbiol. 2025 Oct 9;16:1662779. doi: 10.3389/fmicb.2025.1662779 (PMC12557574; doi:10.3389/fmicb.2025.1662779)
Supplement: Supplementary file 5 [file Data_Sheet_5.PDF]

Supplementary Figure S5

(A)

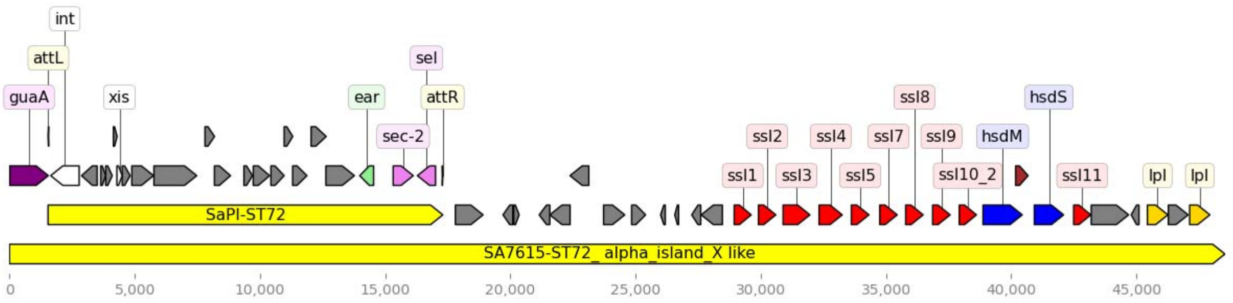

(B)

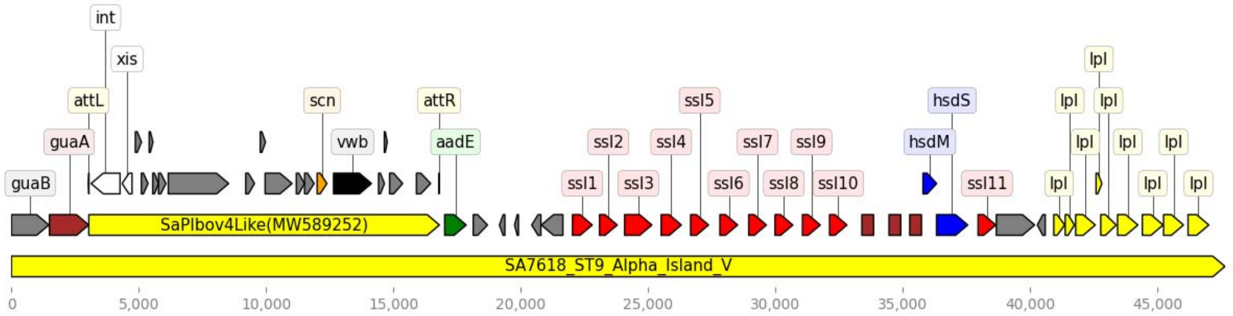

(C)

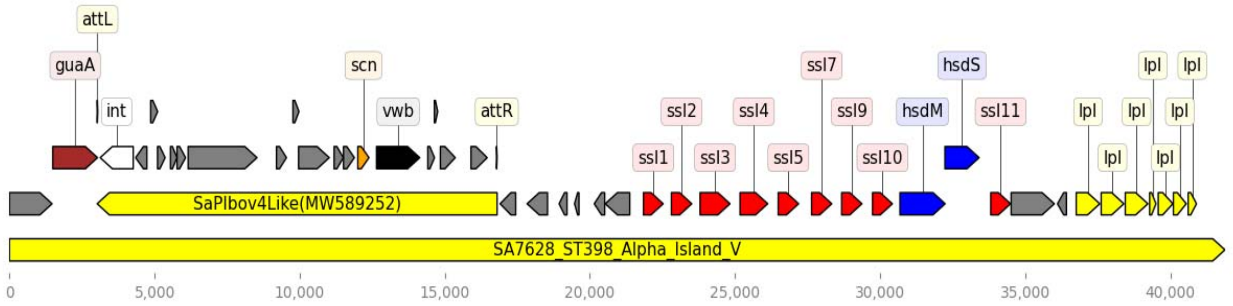

Supplementary Figure S5 (A, B, C) Legend: Comparative structural analysis of the νSaα genomic island in Argentine pig–environment MRSA isolates and reference νSaα types.
(A) νSaα from ST72 isolate SA7615 compared with type X νSaα from *S. aureus* H-EMRSA-15 (NZ_CP007659).
(B) νSaα from ST9 isolate SA7618 compared with type V νSaα from *S. aureus* BA01611 (CP019945.1; ST9).
(C) νSaα from ST398 isolate SA7628 compared with type V νSaα from *S. aureus* DG29 (ST398).
Comparisons were performed using Mauve v2.4.0 (Darling et al., 2004; Rissman et al., 2009) and custom scripts; figures were generated with DNA Features Viewer (https://www.biorxiv.org/content/10.1101/2020.01.09.900589v1.
Open reading frames (ORFs) are shown as arrows indicating transcriptional direction, colored by functional category: white, *int* and *xis*; light green, *ear* (penicillin-binding protein fragment); pink, *sec-2* and *sel*; orange, *scn*; black, *vwb*; green, *aadE*; blue, *hsdS*/*hsdM*; red, *ssl* (staphylococcal superantigen-like) genes; yellow, *lpl* genes; grey, hypothetical proteins.
In panel A, *hsdS* showed 70% identity and *ssl* genes >85% (*ssl11* 75%); all other genes in all panels displayed >95% nucleotide identity with the reference.
The novel ST2 νSaα structure was present in several public ST72 genomes (e.g., CP030550.1, CP062419.1, CP062412.1, CP062402.1, CP062362.1, CP075580.1, CP075581.1, CP075582.1, CP051915.1, CP049384.1, CP029681.1, CP029649.1, CP062416.1, CP062414.1, CP051919.1, CP030558.1), is proposed as a 'νSaα variant type X-like', associated with ST72
.
