# Supplementary material for: Phylogenomic insights into LA-MRSA from Argentine pig farm environments: novel OptrA variant and regional emergence of an ST9 lineage co-circulating with international CC398 lineages
Source: Front Microbiol. 2025 Oct 9;16:1662779. doi: 10.3389/fmicb.2025.1662779 (PMC12557574; doi:10.3389/fmicb.2025.1662779)
Supplement: Supplementary file 6 [file Data_Sheet_6.PDF]

Supplementary Material Data 1

1- Genomic Analyses of Antimicrobial Resistance Genes (ARGs), Mobile Genetic Elements (MGEs), and Virulence Genes in Assembled Genomes.

ARGs and *fosB* subtypes were identified using CGE ResFinder (Bortolaia *et* al., 2020) (accessed 18 August 2024), Pathogenwatch, and the Comprehensive Antibiotic Resistance Database (CARD) (Alcock et al., 2020). OptrA variants were defined as per Schwarz et al. (2021) using WP_063854496.1 as reference. The mobile genetic elements (MGEs) were identified with PlasmidFinder (Carattoli *et al.*, 2014;Carattoli and Hasman, 2020), MobileElementFinder (Johansson et al., 2021), and mapped and visualized with **Proksee** (Grant *et al.*, 2023). Replicase families were defined according to Jensen et al. (2010) and expanded by Lozano et al. (2012). Genomic locations (chromosomal vs plasmid) were inferred using the online tool RFPlasmid (van der Graaf-van Bloois et al., 2021). Virulence genes were detected using VFDB (Liu et al., 2022) and VirulenceFinder v2.0 (Joensen et al., 2014) (accessed 15 September 2024), with confirmation by BLASTn. Comparative analyses of SCC*mec* (IIIA-like, V/A1B1-like) and vSa islands (vSaα, vSaβ) were conducted using Mauve v2.4.0 (Darling et al., 2004;Rissman et al., 2009) and custom scripts. Figures were generated using DNA Features Viewer[2]. SCC*mec* elements were identified by BLASTn against an extended SCC*mec*Finder database; fragmented elements were resolved using full assemblies and high-identity mapping (>80%, ≥1000 bp). SCC*mec* variants were further characterized by virtual hybridization using PCR primers targeting the SCC*mec* IIIA element of strain JCSC1716 (Chongtrakool et al., 2006) for SCC*mec* IIIA-like and array probes to differentiate SCC*mec* variants in CC398-MRSA (Monecke *et al.*, 2018) along with sequence regions defining structures A–H in the JCSC6944 SCC*mec* element (Li et al., 2011) for SCC*mec* V-like. In addition, the primer binding sites used in the SCC*mec* typing by PCR (Chongtrakool et al., 2006) also were detected. vSaβ islands were typed based on ≥90% identity with references (Klaui et al., 2019).

[2] https://www.biorxiv.org/content/10.1101/2020.01.09.900589v1

2-Phylogenetic analysis

Three maximum likelihood (ML) trees were constructed based on core genome SNPs. The first included all 24 MRSA isolates from pig environments, using SA LGA251 (FR821779) as reference. Two lineage-specific ML trees (CC398 and ST9/CC1) included our MRSA isolates, public genomes [CC398, n: 98 (LA: 68 and HuA: 30, (Matuszewska et al., 2022); ST9/CC1, n: 67], and additional local genomes (CC398 n: 5 and ST9/CC1 n:4) from Córdoba (2019–2022). The latter were selected from *SA* strains recovered in ongoing, unpublished One Health surveillance studies (humans, rivers, animals, and food) in central Argentina (Supplementary Table S2.2).

Core SNPs were identified using the approach by Aanensen et al. (2016), based on 26 annotated reference genomes. Briefly, short-reads from our isolates and the 26 public genomes, were mapped to lineage-specific references (LGA251, S0385/ST398, QD-CD9/ST9) using BWA (Li and Durbin, 2009) and processed with SAMtools (Li et al., 2009). Variant calling was performed with **GATK** (McKenna et al., 2010), and **SnpEff** (Cingolani et al., 2012). Unmapped reads and sequences not present in all genomes were excluded from the core genome, and SNPs within mobile genetic elements (MGEs) were also removed. A multiple sequence alignment (MSA) of SNPs was generated using **VcfKit** (Cook and Andersen, 2017) ("phylo fasta" command), excluding

indels. ML trees were inferred using RAxML v4.0 (Stamatakis, 2014, 2015) (GTRCAT model, 1000 bootstraps).

To explore farm-level transmission, two additional SNP trees (CC398 and ST9/CC1, n: 11 each) were generated with CSI Phylogeny v1.4 (Kaas *et al.*, 2014) with lineage-specific reference genomes (S0385/ST398 and QD-CD92/ST9). Pairwise alignment percentages were visualized via heatmaps and dendrograms using ggplot2(v3.4.4) and ComplexHeatmap/(v2.15.4) packages in R Studio R. Given the lack of consensus on SNP cut-offs for transmission inference (<15 to <50 SNPs), a 40-SNP threshold was used to define closely related strains or transmission cluster (Price et al., 2014;Long et al., 2014;Schurch et al., 2018;Bouchami et al., 2020;Randad et al., 2021)
