# Supplementary material for: Phylogenomic insights into LA-MRSA from Argentine pig farm environments: novel OptrA variant and regional emergence of an ST9 lineage co-circulating with international CC398 lineages
Source: Front Microbiol. 2025 Oct 9;16:1662779. doi: 10.3389/fmicb.2025.1662779 (PMC12557574; doi:10.3389/fmicb.2025.1662779)
Supplement: Supplementary file 9 [file Table_3.DOCX]

**Supplementary Table S3.** Antimicrobial use on farms during the 6 months preceding the visit by the study veterinarians.

| Drug Family | Antimicrobial^a^ | ID Farms ^b^ | | | | | | | | | |
| --- | --- | --- | --- | --- | --- | --- | --- | --- | --- | --- | --- |
|  |  | 1* | 2* | 3* | 4* | 5* | 6* | 7* | 8 | 9* | 10 |
| β-lactams | Penicillin | + | + | + | + | + | + |  |  |  | + |
|  | Amoxicillin | + | + | + | + | + | + | + | + | + | + |
|  | Ceftiofur | + | + | + | + | + | + | + | + | + | + |
| Macrolides | Gamithromycin | + | + |  |  |  |  |  |  |  | + |
|  | Tilmicosin | + |  | + |  | + | + |  |  | + | + |
|  | Tylosin | + | + | + | + | + | + | + | + | + | + |
|  | Tilvalosin |  |  |  | + |  |  |  |  |  |  |
|  | Tulathromycin | + | + | + | + | + |  | + | + |  | + |
| Aminoglycosides | Spectinomycin | + |  |  |  | + |  |  |  |  |  |
|  | Streptomycin |  | + | + | + | + | + |  |  |  | + |
|  | Gentamicin |  |  |  | + | + |  |  |  |  |  |
|  | Neomycin | + | + | + | + |  |  | + | + | + | + |
| Phenicols | Florfenicol | + | + | + | + | + | + | + | + | + |  |
| Fluoroquinolones | Ciprofloxacin | + |  |  | + |  |  |  |  |  | + |
|  | Norfloxacin | + | + | + | + | + | + | + | + |  | + |
|  | Enrofloxacin | + | + | + |  | + | + | + | + |  | + |
| Tetracyclines | Chlortetracycline | + |  | + |  |  |  | + | + |  | + |
|  | Doxycycline |  | + | + |  | + |  |  |  |  | + |
|  | Oxytetracycline | + | + | + |  |  |  |  |  |  | + |
| Fosfomycin | Fosfomycin |  | + |  |  | + | + | + | + |  | + |
| Pleuromutilins | Tiamulin | + |  | + |  |  | + | + | + |  | + |
| Sulfonamides | Sulfadiazine | + | + |  |  |  |  |  |  |  | + |
| Lincosamides | Lincomycin | + |  |  |  | + | + |  |  |  |  |

**^a^** “+” indicates that the antimicrobial was used on the farm during the 6 months preceding the visit by the study veterinarians.
**^b^** MRSA-positive farms are marked with an asterisk (*).
